# Assessment of Grades of Recommendations and Applicability of Royal College of Obstetricians and Gynaecologists Green‐Top Guidelines: A Cross‐Sectional Study

**DOI:** 10.1111/1471-0528.70230

**Published:** 2026-04-01

**Authors:** Anangsha Kumar, Alexandra Cocking, Megan Hall, James Morris, Srividhya Sankaran, Holly Lovell, Andrew Shennan, Kypros Nicolaides, Lisa Story

**Affiliations:** ^1^ Institute of Women and Children's Health, School of Life Course and Population Sciences King's College London London UK; ^2^ Department of Women and Children's Health Guy's and St Thomas' NHS Foundation Trust London UK; ^3^ Department of Obstetrics and Gynaecology Cardiff and Vale University Health Board Cardiff UK; ^4^ Department of Population Health Sciences King's College London London UK; ^5^ Fetal Medicine Research Institute King's College Hospital London UK

**Keywords:** generalisability, guidelines, health equity, obstetric

## Abstract

**Background:**

Royal College of Obstetricians and Gynaecologists (RCOG) Green‐top Guidelines (GTGs) provide evidence‐based recommendations in women's health. Even where evidence is considered high quality, it is uncertain whether factors known to influence maternity outcomes are reflected in study design.

**Objectives:**

To determine distribution of recommendation grades across obstetric GTGs. For Grade A recommendations, to evaluate if supporting studies reported equity‐relevant design features and methodological robustness.

**Design:**

Review of RCOG obstetric GTGs.

**Setting:**

United Kingdom.

**Sample:**

All RCOG non‐archived obstetrics GTGs published up to 20 April 2025.

**Methods:**

Frequencies of Grade A‐D recommendations and Good Practice Points were recorded. For Grade A recommendations, underpinning studies were assessed for health equity and generalisability. All primary studies underpinning every Grade A recommendation were included; where a single study supported multiple recommendations, it was included every time and mapped to each relevant recommendation. The median health equity and generalisability score for each recommendation (if > 1 supporting studies) and a median score of all Grade A recommendations per guideline was calculated.

**Main Measure Outcomes:**

Distribution of recommendation grades: health equity and methodological robustness scores for studies underpinning Grade A recommendations.

**Results:**

Variable frequencies of Grade A, B, C, D recommendations and GPP from 37 eligible guidelines were noted. Only 28 GTGs had Grade A recommendations, with median health equity and generalisability scores of 1 of 13 and 6 of 10 respectively across those recommendations.

**Conclusions:**

Twenty‐four percent of obstetric GTGs have no Grade A recommendations. Of those that do, consideration of health equity and generalisability in associated studies is limited. These design and reporting features should be considered in future research to improve applicability of clinical guidance to all patient groups.

## 1 Introduction

Over recent decades, there has been a growing emphasis on evidence‐based medicine, which involves the conscientious and judicious use of best current evidence to inform clinical practice [1]. Historically, Obstetrics and Gynaecology lagged behind other specialties in integrating robust evidence into clinical practice [2]. However, the introduction of the Royal College of Obstetricians and Gynaecologists (RCOG) Green‐top Guidelines (GTGs) has been instrumental in promoting the use of evidence‐based practices in maternity care.

Recommendations in RCOG GTGs are graded (A‐D or Good Practice Point) based on the quality and type of evidence underpinning them. Grade A is supported by the highest level of evidence, based on high‐quality meta‐analyses, systematic reviews or randomised controlled trials, whereas grade D is considered less robust and based primarily on case studies, series, or expert opinion (Tables S1 and S2) [3].

Recent maternity reports have highlighted significant disparities in pregnancy outcomes for women from ethnic minority backgrounds [4]. Women of Asian and Black descent experience two‐ and three‐fold increased maternal mortality rates (MMR) respectively compared to White women [4]. Maternal mortality is defined as death during pregnancy through six weeks postpartum as described by Mothers and Babies: Reducing Risk Through Audits & Confidential Enquiries Across the United Kingdom (MBRRACE). Women from the most socially deprived areas are disproportionately affected, with the latest MBRRACE report showing that 9% of maternal deaths occurred in women experiencing disadvantages, including mental health conditions, substance use, and domestic abuse [4]. These vulnerabilities are common in pregnant women, with around 20% having a diagnosed mental health condition [5], 5%–10% affected by substance misuse [6] and one in three experiencing domestic abuse [7]. Insufficient support for linguistic needs among women who have recently arrived in the United Kingdom and a lack of consideration for literacy levels, both of which exacerbate poor pregnancy outcomes, have also been highlighted [4]. Although GTGs are likely to have improved patient care in obstetrics, it is unclear if these factors have been considered when recommendations have been made [8].

This study aims to assess RCOG obstetric GTGs to identify the proportion of Grade A recommendations based on high‐level evidence and to evaluate studies underpinning these high‐quality recommendations for consideration of health equity and generalisability in their study design. This assessment does not evaluate downstream equity and generalisability in clinical outcomes and is limited to reporting and design features of underpinning studies.

## 2 Methods

### 2.1 Ethics

This study did not involve human participants or patient data and therefore did not require ethical approval.

### 2.2 Inclusion Criteria

All non‐archived GTGs published by the RCOG were screened for eligibility. Guidelines were classified as obstetric if listed under the ‘Obstetrics’ category on the RCOG GTG website. Guidelines pertaining to gynaecology, as well as those under development as of 20 April 2025, were excluded.

For each GTG, the total number of recommendations (A–D, and those considered Good Practice Points) was recorded (Figure S1). For each Grade A recommendation, all supporting Level 1++ and 1+ studies explicitly cited within the GTG evidence base were systematically extracted. Where a single Grade A recommendation was informed by multiple studies, all eligible studies were included in the assessment (Figure S2). These supporting studies were then evaluated for consideration of health equity and generalisability using two scoring systems: a de novo framework for assessing health equity, and a validated framework for appraising generalisability.

### 2.3 Health Equity

A maternity‐specific Health Equity screening tool was developed by the study team. This tool was informed by factors identified in the MBRRACE‐UK 2024 ‘Saving Lives, Improving Mothers’ Care’ report, which were known to be associated with an increased risk of maternal mortality in the United Kingdom between 2020 and 2022 [4]. Ten factors were included: age, race/ethnicity and culture (single factor), disability, language barriers, socioeconomic status, deprivation index, vulnerability, occupation, migrancy status, and body mass index (BMI). The tool was further enhanced by addition of three extra parameters identified from two international health equity frameworks, Health Equity Assessment Toolkit (HEAT) by WHO [9] and the PROGRESS‐PLUS framework by the Cochrane and Campbell Equity Methods Group [10]. These were sexual orientation, religion and educational attainment.

HEAT comprises of eleven parameters; age, sex, race, religion, disability, sexual orientation, pregnancy, marriage, economic status, place of residence, and vulnerable groups; and PROGRESS‐PLUS includes: age, sex, race, ethnicity, culture, language, religion, disability, socioeconomic status, education, place of residence, social capital, and occupation, features of relationship (e.g., smoking parents, excluded from school) and time dependant relationship (leaving the hospital, respite care, other instances where a person may be temporarily at a disadvantage). Six domains within these two frameworks were not included: marriage—as no studies were identified that had explored the role of marital status on maternal outcomes after accounting for socioeconomic status [11]; pregnancy—considering the focus of this work was on obstetric guidelines; social capital—because it is difficult to measure consistently and overlaps conceptually with vulnerability and socioeconomic status; sex—as the tool is specific to maternity care and therefore inherently limited to those who are pregnant; features of relationship—as these are primarily relevant to child and adolescent health and have limited applicability to maternal outcomes; and time‐dependent relationships—as they represent transient vulnerabilities rather than enduring determinants of maternal health inequity. The rest of the parameters were overlapping with the ten factors already identified from MBRRACE.

The final screening tool therefore comprised 13 predefined domains mutually agreed among all the authors, each scored dichotomously (0 = not considered; 1 = explicitly considered) based on study design or reporting (Table S3). The total score out of 13 therefore represents the number of predefined equity domains considered in the studies, not the relative importance of the studies nor the magnitude/direction of inequity. The maximum possible score was 13; however, observed ranges reflect the number of equity domains reported by studies. Reported score ranges reflect variability between underpinning studies supporting the same Grade A recommendation, rather than uncertainty in scoring. Median scores were used to summarise overall performance, where multiple studies were involved for a recommendation or multiple recommendations existed for a single GTG.

For the purposes of this screening tool, migrancy, place of residence, and occupation were considered to be addressed if the study reported any explicit descriptor at baseline (e.g., country of birth or migration status; rural/urban/remote location or area‐based deprivation; or employment status or occupational category), without requiring further disaggregation or analytical use of these variables.

### 2.4 Generalisability

To assess methodological robustness relevant to the interpretability and applicability of the evidence underpinning Grade A recommendations, a validated framework developed by Berger et al. (2009) [12] was used. This incorporates criteria from established methodological appraisal instruments, including the Jadad score [13], Cochrane Back Review Group criteria [14], Delphi list [15], and CONSORT statement [12, 16]. It comprises 10 parameters: randomisation, masking, allocation concealment, handling of withdrawals and dropouts, measures of variability, prespecified analysis including endpoints, stopping rules, statistical methods, baseline data and addressing multiplicity. This framework primarily assesses internal validity and trial conduct; it was not intended to measure external validity or population representativeness. Each parameter was scored (1 or 0), and the resulting scores were summed to give a maximum possible total of 10 (Table S4).

To improve consistency of application, following finalisation of the two scoring frameworks for health equity and generalisability (Tables S3 and S4), two reviewers (AK and AC) independently applied these tools to all eligible studies supporting Grade A recommendations. Inter‐rater reliability was undertaken by calculating Intraclass Correlation Coefficient (ICCs) between these scores given by individual reviewers prior to disagreements being resolved by the third. Eight discrepancies in generalisability scoring and five in equity scoring were resolved by a third reviewer (LS).

## 3 Results

Seventy‐seven unarchived RCOG Green‐top guidelines were initially identified. Eighteen gynaecology guidelines were excluded. Of the remaining 59 obstetric guidelines, after accounting for ‘duplicates’ (*n* = 21) and guidelines still in development (*n* = 1), the final dataset comprised of 37 obstetric guidelines (Figure S1). Of the obstetric guidelines identified, some appeared more than once on the RCOG website because the same guideline was listed under multiple obstetric subcategories. These ‘duplicate’ listings (*n* = 21 as above) (e.g., *Epilepsy in Pregnancy* listed under both Antenatal Care and Pre‐existing Medical Conditions, and *Maternal Collapse in Pregnancy* listed under Antenatal Care and Labour and Birth) were counted once, with a single version retained for analysis. In total 1848 recommendations were made; of which 5.3% were Grade A (*n* = 99), 11.2% were Grade B, 14.8% were Grade C, 25.4% were Grade D and 43.2% were GPP. The frequency of recommendation grades for each guideline can be seen in Table S5.

Nine guidelines (24.3% of eligible obstetric guidelines) had no grade A recommendations. The guideline most reliant on low grade recommendations was GTG No. 12 ‘Pregnancy and Breast Cancer’ which comprised 5% Level C, 20% Level D and 75% GPP recommendations. Of the guidelines with Grade A recommendations, GTG No. 73 ‘Care of Women Presenting with Suspected Preterm Prelabour Rupture of Membranes from 24 + 0 Weeks of Gestation’ had the highest proportion of Grade A recommendations (30%), (Table S5).

The health equity and generalisability scores for 99 Grade A recommendations across 28 guidelines are presented in Table S6 with underpinning studies for each recommendation reported in Table S7. ICCs between the study‐level scores by individual reviewers prior to disagreements being resolved showed excellent reliability (ICC for Equity scores: 0.988 with 95% CI: 0.984–0.991 and *p* < 0.001; ICC for Generalisability scores: 0.994 with 95% CI: 0.992–0.995 and *p* < 0.001). The equity tool had a maximum possible score of 13, observed scores across 99 recommendations ranged from 0 to 4 with a median score of 1 across these recommendations, indicating a marked floor effect. Most studies addressed only one or two equity domains, with no study reporting more than four domains. Hence, the distribution of equity scores was highly skewed towards lower values, with the majority of studies scoring 0 or 1 (Table S6). Most studies considered age and BMI, but very few addressed key determinants such as race/ethnicity, socioeconomic status, disability, or language needs, and none reported religion [17], highlighting gaps in reporting and study design (Table S7). Similarly, across 99 Grade A recommendations, the median generalisability score was 6 out of 10 (range 0–9).

Across 28 guidelines the median health equity score was 0.5 (range 0–4) whereas the median generalisability score was 5 (range 0–8), with some guidelines having more than one Grade A recommendation. GTG No 64 ‘Management of maternal sepsis during and following pregnancy’ achieved the highest health equity score of 4 based on one high quality study. Eight GTGs (seven with > 1 Grade A recommendation) had a median score of 0 for health equity (Table S8). GTG No. 75 ‘Cervical Cerclage’ gained the highest generalisability score with a median score of 8 (range 7–9) comprising two Grade A recommendations, each based on a single study. GTG 45 ‘Birth after Previous Caesarean Section’ and GTG No. 64 ‘Management of maternal sepsis during and following pregnancy’ scored lowest (0) for generalisability (Table S9). Therefore, when summarised at guideline level, median scores reflected aggregation of scores of multiple Grade A recommendations within that individual guideline with a final median score across all eligible guidelines; whereas when summarised at recommendation level, the scores represented an aggregation of scores from the various underpinning high‐quality studies with a final median score across all 99 recommendations.

## 4 Discussion

### 4.1 Main Findings

This analysis of obstetric RCOG GTGs highlights that a high proportion (24.32%) of current guidelines do not include recommendations underpinned by highest quality of evidence. Of guidelines that do, these still represent a small proportion (5.36%) of total recommendations. We have demonstrated considerable variation in distribution of grades of recommendations across obstetric guidelines ranging from 0% to 30% for Grade A, 0%–45.5% for Grade B, 0%–35.6% for Grade C, 0%–61.5% for Grade D and 0%–75% for GPP. Consideration and reporting of health equity and generalisability relevant domains is limited, even where evidence is of highest quality with equity scores ranging from 0 to 4 and generalisability scores ranging from 0 to 8 at a guideline level.

### 4.2 Interpretation

Several reasons underlie the finding that many recommendations are not based on highest levels of evidence. Firstly, disease prevalence may affect number of studies on which recommendations can be based, for example, GTG No. 12 ‘Pregnancy and Breast Cancer’. Although an important issue, incidence of new diagnosis of breast cancer during pregnancy affects 0.03% of pregnant women [18], therefore the feasibility of randomised trials and meta‐analyses are limited. Moreover, maternity is generally underrepresented in allocations of grant funding, impacting the frequency of large well‐designed studies underpinning high grade recommendations [19]. Additionally, practices such as Cervical Cerclage have historically become embedded into routine care without being rigorously evaluated by clinical trials, prior to implementation [20].

Our analysis also shows that health equity and generalisability are poorly considered in studies forming the basis of Grade A recommendations. Although other groups have assessed evidence grades and their quality for RCOG GTGs [21, 22], none to our knowledge have additionally assessed health equity and methodological robustness of underpinning studies. This is important as certain obstetric complications are more prevalent in specific groups, as highlighted in MBRRACE‐UK, which found higher overall mortality in Black and Asian women [4]. Specific conditions, where this is particularly relevant include post‐partum haemorrhage (PPH). Recent research demonstrated a higher incidence of PPH and associated maternal morbidity in Black women and those in deprived areas [23, 24, 25]. Findings from our study indicated that these factors were not comprehensively considered in studies underpinning Grade A recommendations in the RCOG GTG No. 52 ‘Prevention and Management of Postpartum Haemorrhage’, which had a median score of 0.5 of 13 for health equity. This GTG has four Grade A recommendations, each supported by one high‐quality evidence, but only one of those four scored for ethnicity. Maternal thromboembolic disease is another example, where disease prevalence is known to alter by ethnic group [26]. Despite these findings, GTG No. 37a ‘Reducing the Risk of Venous Thrombo‐Embolism (VTE) during Pregnancy and the Puerperium’ scored poorly for equity. Of its two Grade A recommendations, one was based on seven studies and the other on one, but all scored zero for equity.

Our finding, that health equity is not routinely considered in study design is consistent with other maternity research [8, 27, 28]. One contributing factor to this finding is that disparities in outcomes has only been highlighted in more recent maternity reports. The first United Kingdom maternal mortality report highlighting this was published in 2004 [29]. More than one fifth (21.2%) of the studies underpinning Grade A recommendations identified in our study were published prior to this, with one dating as far back as 1978 [30]. Additionally, in recent decades, the UK obstetric population has significantly changed, increasing in medical and social complexity, impacting translatability of findings from older studies. Although lack of equity in study design is prevalent [8], it should also be noted that some guidelines are specific to certain populations and therefore all the parameters of our toolkit are not relevant. For example, GTG No. 66 ‘Management of Beta Thalassaemia in Pregnancy’, where ethnic diversity in underpinning research is less relevant, as the condition is strongly associated with certain ethnic groups.

Lack of diversity in research is not limited to maternity; it has been widely raised as a scientific and medical issue [31]. Early drug trials for antihypertensive and anti‐platelet agents did not consider that different ethnicities may have different responses to medications. Subsequent research has indicated they are not equally efficacious in all groups [32]. To derive translatable results, study populations must reflect groups affected by the conditions being investigated [33]. Such lessons must be embraced by all specialties when designing trials. Additionally, rather than proposing a fixed toolkit, it is essential to emphasise the importance of context‐sensitive and ethically governed reporting of equity‐relevant characteristics, recognising that the implications of collecting such data may vary across settings.

We found wide variation in generalisability across evidence underpinning obstetric GTGs recommendations, reflecting the long‐standing methodological issues in maternity research design [34, 35]. Our finding is also consistent with previous work, addressing the quality of evidence underpinning RCOG guidance from 2014 [22], which also found that guideline development often proceeds without strong empirical data. While guidelines remain essential for standardising care, the predominance of low‐quality evidence requires acknowledgement, especially where recommendations rely on expert consensus rather than trial data [22].

### 4.3 Strengths and Limitations

This analysis evaluates study design and reporting characteristics rather than the real‐world impact of guideline implementation. We used a systematic approach to assess all current obstetric RCOG GTGs, providing up‐to‐date evaluation of the evidence base underpinning clinical recommendations. The use of two scoring frameworks ensures a structured appraisal of the quality of supporting evidence. Independent dual review, with discrepancies resolved by a third reviewer, minimised bias. Additionally, this is the first study, to our knowledge, to evaluate both equity and generalisability in the underpinning evidence of high‐quality obstetric guideline recommendations.

The analysis was restricted to studies cited in Grade A recommendations; therefore, recommendations supported by lower‐level evidence were not assessed for equity, though they comprised most of the guidance. The generalisability scoring system was only suitable for specific study types, limiting its applicability to screen low quality evidence. As with all secondary analyses, results depended on the accuracy of reporting in the original studies, which may have introduced bias. While our findings highlight gaps in the evidence base, they do not directly evaluate the impact of guideline use on outcomes.

Additionally, the equity tool was developed for screening and descriptive appraisal and is therefore not a psychometrically validated measurement instrument. Therefore, resulting scores should not be interpreted as a validated metric of equity, but as a transparent, reproducible screening approach aligned with recognised equity frameworks. Further work is required to formally evaluate reliability, construct validity, and weighting of domains. It was not intended to quantify equity, but to identify whether key equity‐related domains were explicitly considered in the design or reporting of studies. Collection of migrancy‐related variables also raises ethical considerations, as in some contexts such data may be perceived as threatening and could discourage participation among socioeconomically vulnerable groups; this analysis evaluates reporting only and does not advocate indiscriminate data collection.

Although referred to as generalisability in this study, the Berger framework primarily assesses internal validity and trial quality rather than external validity. It does not capture population representativeness or setting‐specific applicability; therefore, findings should be interpreted as reflecting methodological robustness.

## 5 Recommendations

This study demonstrates that while RCOG guidelines provide a valuable framework, there remains a gap in high‐quality research in obstetrics supporting their recommendations. Future studies should integrate health equity and generalisability into their design to ensure applicability across diverse maternity populations. Guideline review processes should incorporate explicit appraisal of these parameters using established tools when assessing evidence quality, enabling recommendations that are both methodologically robust and equitable in scope and be clear when the evidence is lacking to enable clinicians apply them alongside clinical judgement considering local resources [12]. To support meaningful equity appraisal, future obstetric trials should report a minimum core set of demographic characteristics, including age, ethnicity, socioeconomic indicators, place of residence, migrancy status (where appropriate), disability, and occupation. For occupation, reporting of working conditions such as physical demands, job insecurity, or exposure to occupational hazards would improve interpretability. Such reporting should be guided by ethical principles to avoid unintended harms. Future research should ensure that equity‐related data are collected with appropriate safeguards, transparency, and community engagement, and only where clearly justified and ethically governed.

## 6 Conclusion

This study highlights substantial gaps in generalisability and equity considerations within the evidence underpinning obstetric GTGs, highlighting the need for targeted research investment, consistent use of equity‐focused frameworks, and deliberate recruitment strategies to ensure future guidelines are underpinned by methodologically robust evidence that more comprehensively considers equity‐relevant population characteristics.

## Author Contributions

Anangsha Kumar – Data curation, Validation, Methodology, Writing: original draft, Writing: review and editing, Alexandra Cocking – Methodology, James Morris – Data curation, Srividhya Sankaran – Writing: review and editing, Holly Lovell – Methodology, Writing: review and editing, Andrew Shennan – Writing: review and editing, Kypros Nicolaides – Writing: review and editing; Lisa Story – Visualisation, Supervision, Writing: review and editing.

## Funding

Dr. Lisa Story is funded by an NIHR Advanced Fellowship [NIHR30166]. Ms. Holly Lovell is funded by an NIHR Doctoral Clinical Academic Fellowship [NIHR302860].

## Conflicts of Interest

The authors declare no conflicts of interest.

## Supporting information


**Figure S1:** Flow diagram of RCOG Green‐top guideline selection and analysis.
**Figure S2:** Types of included studies.

**Table S1:** RCOG system for classifying evidence (Table adapted from Developing a Green‐top Guideline by RCOG).
**Table S2:** RCOG system for classifying grading recommendations (Table adapted from Developing a Green‐top Guideline by RCOG).
**Table S3:** Green Top Guideline Health Equity Scoring Criteria (Each criterion scored 0 or 1, with a maximum of 1 point per criterion).
**Table S4:** Green Top Guideline Generalisability Scoring Criteria (Each criterion scored 0 or 1, with a maximum of 1 point per criterion).
**Table S5:** Frequencies of grading of recommendations in Obstetric Green top Guidelines.
**Table S6:** Median score for evidence underpinning each Grade A recommendations (recommendation Level). Ranges differ by level of aggregation (recommendation‐level vs. guideline‐level medians).
**Table S7:** Underpinning studies for Grade A recommendations.
**Table S8:** Median health equity score for each guideline (guideline level). Ranges differ by level of aggregation (study‐level vs. guideline‐level medians).
**Table S9:** Median generalisability score for each guideline. Ranges differ by level of aggregation (study‐level vs. guideline‐level medians).

## Data Availability

The data that supports the findings of this study are available in the Supporting Information of this article.
