# Supplementary material for: Assessment of Grades of Recommendations and Applicability of Royal College of Obstetricians and Gynaecologists Green‐Top Guidelines: A Cross‐Sectional Study
Source: BJOG. 2026 Apr 1;133(9):1771–6. doi: 10.1111/1471-0528.70230 (PMC13419000; doi:10.1111/1471-0528.70230)
Supplement: Supplementary file 1 — Figure S1: Flow diagram of RCOG Green‐top guideline selection and analysis. Figure S2: Types of included studies. [file BJO-133-1771-s001.docx]

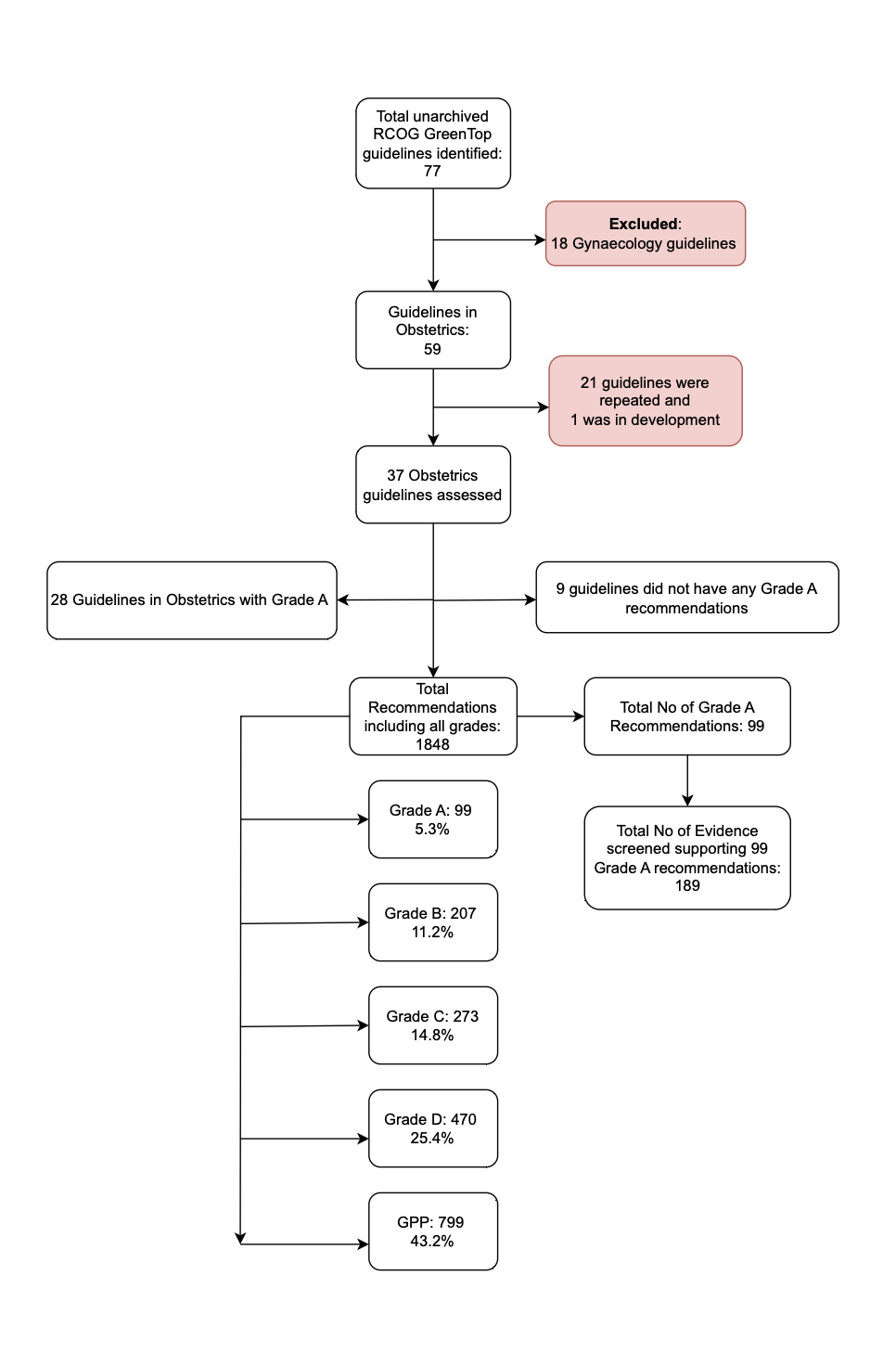
Figure S1: Flow diagram of RCOG Green-top guideline selection and analysis

*
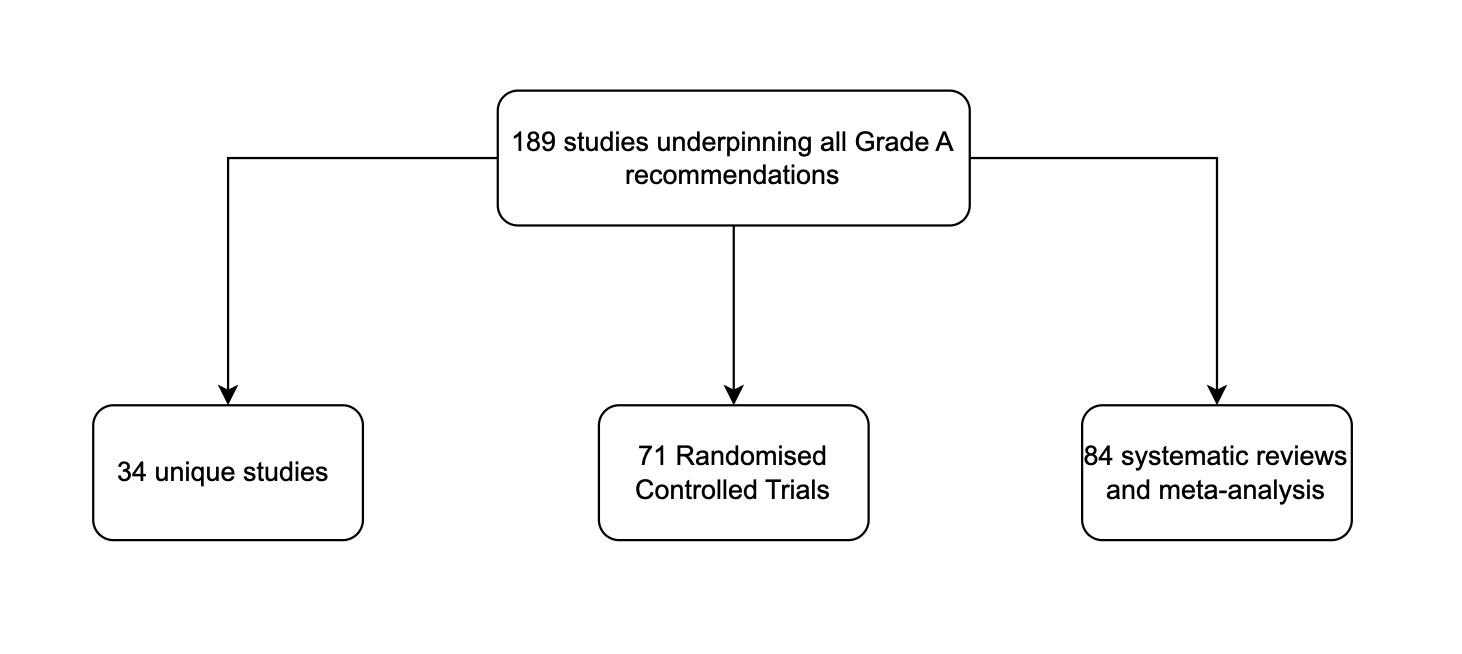
*

Figure S2: Types of included studies
