# Supplementary material for: Assessment of Grades of Recommendations and Applicability of Royal College of Obstetricians and Gynaecologists Green‐Top Guidelines: A Cross‐Sectional Study
Source: BJOG. 2026 Apr 1;133(9):1771–6. doi: 10.1111/1471-0528.70230 (PMC13419000; doi:10.1111/1471-0528.70230)
Supplement: Supplementary file 2 — Table S1: RCOG system for classifying evidence (Table adapted from Developing a Green‐top Guideline by RCOG). Table S2: RCOG system for classifying grading recommendations (Table adapted from Developing a Green‐top Guideline by RCOG). Table S3: Green Top Guideline Health Equity Scoring Criteria (Each criterion scored 0 or 1, with a maximum of 1 point per criterion). Table S4: Green Top Guideline Generalisability Scoring Criteria (Each criterion scored 0 or 1, with a maximum of 1 point per criterion). Table S5: Frequencies of grading of recommendations in Obstetric Green top Guidelines. Table S6: Median score for evidence underpinning each Grade A recommendations (recommendation Level). Ranges differ by level of aggregation (recommendation‐level vs. guideline‐level medians). Table S7: Underpinning studies for Grade A recommendations. Table S8: Median health equity score for each guideline (guideline level). Ranges differ by level of aggregation (study‐level vs. guideline‐level medians). Table S9: Median generalisability score for each guideline. Ranges differ by level of aggregation (study‐level vs. guideline‐level medians). [file BJO-133-1771-s002.docx]

| **Classification of evidence levels** | **Description of evidence levels** |
| --- | --- |
| **1++** | High quality meta-analyses, systematic reviews of randomised controlled trials or randomised controlled trials with a very low risk of bias |
| **1+** | Well-conducted meta-analyses, systematic reviews of randomised controlled trials or randomised controlled trials with a low risk of bias |
| **1-** | Meta-analyses, systematic reviews of randomised controlled trials or randomised controlled trials with a high risk of bias |
| **2++** | High-quality systematic reviews of case–control or cohort studies or high-quality case–control or cohort studies with a very low risk of confounding, bias or chance and a high probability that the relationship is causal |
| **2+** | Well-conducted case–control or cohort studies with a low risk of confounding, bias or chance and a moderate probability that the relationship is causal |
| **2-** | Case–control or cohort studies with a high risk of confounding, bias or chance and a significant risk that the relationship is not causal |
| **3** | Non-analytical studies, e.g. case reports, case series |
| **4** | Expert opinion |

Table S1: RCOG system for classifying evidence (Table adapted from Developing a Green-top Guideline by RCOG)

| **Grades of recommendations** | **Description of grades of recommendations** |
| --- | --- |
| **A** | At least one meta-analysis, systematic review or randomised controlled trial rated as 1++ and directly applicable to the target population; or  A systematic review of randomised controlled trials or a body of evidence consisting principally of studies rated as 1+ directly applicable to the target population and demonstrating overall consistency of results |
| **B** | A body of evidence including studies rated as 2++ directly applicable to the target population and demonstrating overall consistency of results; or  Extrapolated evidence from studies rated as 1++ or 1+ |
| **C** | A body of evidence including studies rated as 2+ directly applicable to the target population and demonstrating overall consistency of results; or  Extrapolated evidence from studies rated as 2++ |
| **D** | Evidence level 3 or 4; or  Extrapolated evidence from studies rated as 2+ |
| **Good practice point** | Recommended best practice based on the clinical experience of the guideline development group |

Table S2: RCOG system for classifying grading recommendations ((Table adapted from Developing a Green-top Guideline by RCOG)

| **Criteria Number** | **Green Top Guideline** **Health Equity Scoring Criteria** | **Origin** |
| --- | --- | --- |
| **1** | Age | MBRRACE, HEAT, PROGRESS-PLUS |
| **2** | Race, Ethnicity, Culture | MBRRACE, HEAT, PROGRESS-PLUS |
| **3** | Religion | HEAT/PROGRESS-PLUS |
| **4** | Disability (Eg: Physical, Intellectual, Mental Health) | MBRRACE, HEAT, PROGRESS-PLUS |
| **5** | Sexual Orientation (Heterosexual, Homosexual, Bisexual, Other, Gender Reassignment) | HEAT/PROGRESS-PLUS |
| **6** | Language Barrier | MBRRACE, PROGRESS-PLUS |
| **7** | Vulnerable Groups - (Eg: Substance Misuse, Domestic Abuse, History of Abuse as a child) | MBRRACE, HEAT |
| **8** | Migrants | MBRRACE |
| **9** | Education (Nil, Primary, Secondary, Tertiary) | HEAT/PROGRESS-PLUS |
| **10** | Socioeconomic Status (Income Level, Wealth, Housing Status) | MBRRACE, HEAT, PROGRESS-PLUS |
| **11** | Place of Residence (Eg: Urban, Rural, Remote) | MBRRACE, HEAT, PROGRESS-PLUS |
| **12** | Occupation (Eg: Employment Status, Job Type, Working Conditions) | MBRRACE, PROGRESS-PLUS |
| **13** | Body Mass Index | MBRRACE |

Table S3: Green Top Guideline Health Equity Scoring Criteria (Each criterion scored 0 or 1, with a maximum of 1 point per criterion)

| **Criteria Number** | **Green Top Guideline Generalisability Scoring Criteria** |
| --- | --- |
| **1** | Randomisation |
| **2** | Masking |
| **3** | Allocation Concealment |
| **4** | Withdrawal and Dropouts |
| **5** | Measures of Variability |
| **6** | Prespecified Analysis – Endpoints |
| **7** | Stopping Rules |
| **8** | Statistical Methods |
| **9** | Baseline Data |
| **10** | Address Multiplicity |

Table S4: Green Top Guideline Generalisability Scoring Criteria (Each criterion scored 0 or 1, with a maximum of 1 point per criterion)

| **Number** | **Guideline Name** | **number of recommendations** | **% A** | **% B** | **% C** | **% D** | **% GPP** |
| --- | --- | --- | --- | --- | --- | --- | --- |
| 1 | Care of Women Presenting with Suspected Preterm Prelabour Rupture of Membranes from 24+0 Weeks of Gestation - June 2019 | 20 | 30.0% | 20.0% | 0.0% | 20.0% | 30.0% |
| 2 | Malaria in Pregnancy, Prevention - April 2010 | 11 | 27.3% | 45.5% | 18.2% | 9.1% | 0.0% |
| 3 | Intrahepatic cholestasis of pregnancy - June 2022 | 39 | 23.1% | 2.6% | 7.7% | 61.5% | 5.1% |
| 4 | The Diagnosis and Treatment of Malaria in Pregnancy - April 2010 | 33 | 18.2% | 27.3% | 18.2% | 12.1% | 24.2% |
| 5 | Assisted Vaginal Birth - April 2020 | 74 | 17.6% | 14.9% | 9.5% | 12.2% | 45.9% |
| 6 | External Cephalic Version and Reducing the Incidence of Term Breech Presentation - March 2017 | 23 | 13.0% | 34.8% | 21.7% | 4.3% | 26.1% |
| 7 | Antenatal corticosteroids to reduce neonatal morbidity and mortality - October 2010 | 31 | 9.7% | 29.0% | 6.5% | 32.3% | 22.6% |
| 8 | Investigation and Care of a Small-for-Gestational-Age Fetus and a Growth Restricted Fetus - May 2024 | 85 | 7.8% | 7.8% | 13.7% | 31.4% | 39.2% |
| 9 | Prevention and Management of Postpartum Haemorrhage - December 2016 | 51 | 7.3% | 19.5% | 19.5% | 29.3% | 24.4% |
| 10 | The Management of Nausea and Vomiting in Pregnancy and Hyperemesis Gravidarum – February 2024 | 41 | 8.2% | 22.4% | 22.4% | 2.4% | 44.7% |
| 11 | Management of Breech Presentation – March 2017 | 43 | 7.0% | 11.6% | 32.6% | 11.6% | 37.2% |
| 12 | Care of late intrauterine fetal death and stillbirth – October 2024 | 126 | 6.3% | 9.5% | 14.3% | 23.0% | 46.8% |
| 13 | Management of Monochorionic Twin Pregnancy – 2024 Partial Update | 63 | 6.3% | 6.3% | 15.9% | 25.4% | 46.0% |
| 14 | Amniocentesis and Chorionic Villous sampling - December 2024 | 18 | 5.6% | 16.7% | 27.8% | 11.1% | 38.9% |
| 15 | The Management of Third- and Fourth-Degree Perineal Tears – June 2015 | 37 | 5.4% | 13.5% | 10.8% | 21.6% | 48.6% |
| 16 | Management of Thyroid Disorders in Pregnancy – June 2025 | 59 | 5.1% | 20.3% | 35.6% | 30.5% | 8.5% |
| 17 | Care of Women with Obesity in Pregnancy – November 2018 | 64 | 4.7% | 18.8% | 7.8% | 28.1% | 40.6% |
| 18 | Management of Beta Thalassaemia in Pregnancy – March 2014 | 49 | 4.1% | 4.1% | 8.2% | 12.2% | 71.4% |
| 19 | Blood Transfusion in Obstetrics – May 2015 | 49 | 4.1% | 6.1% | 14.3% | 49.0% | 26.5% |
| 20 | Antepartum haemorrhage – November 2011 | 55 | 3.6% | 3.6% | 9.1% | 20.0% | 63.6% |
| 21 | Cervical Cerclage – February 2025 | 58 | 3.4% | 22.4% | 17.2% | 19.0% | 37.9% |
| 22 | Reduced Fetal Movements – February 2011 | 31 | 3.2% | 32.3% | 29.0% | 0.0% | 35.5% |
| 23 | Maternal Collapse in Pregnancy and the Puerperium – December 2024 | 64 | 3.1% | 3.1% | 15.6% | 15.6% | 62.5% |
| 24 | Birth after Previous Caesarean Section – October 2015 | 32 | 3.1% | 21.9% | 18.8% | 18.8% | 37.5% |
| 25 | Prevention of Early-onset Group B Streptococcal Disease – September 2017 | 39 | 2.6% | 10.3% | 28.2% | 28.2% | 30.8% |
| 26 | Reducing the Risk of VTE during Pregnancy and the Puerperium – April 2015 | 79 | 2.5% | 7.6% | 19.0% | 32.9% | 38.0% |
| 27 | Identification and management of maternal sepsis during and following pregnancy – December 2024 | 41 | 2.4% | 0.0% | 2.4% | 26.8% | 68.3% |
| 28 | Management of Inherited Bleeding Disorders – April 2017 | 163 | 1.2% | 6.1% | 9.8% | 35.0% | 47.9% |
| 29 | Pregnancy and Breast Cancer – March 2011 | 20 | 0.0% | 0.0% | 5.0% | 20.0% | 75.0% |
| 30 | Chicken Pox in Pregnancy – Minor Update 2024 | 34 | 0.0% | 0.0% | 11.8% | 38.2% | 50.0% |
| 31 | Epilepsy in pregnancy – June 2016 | 70 | 0.0% | 5.7% | 22.9% | 20.0% | 51.4% |
| 32 | Female Genital Mutilation and it's management – July 2015 | 42 | 0.0% | 0.0% | 2.4% | 28.6% | 69.0% |
| 33 | Placenta Praevia, Placenta Praevia Accreta and Vasa Praevia: Diagnosis and Management – September 2018 | 66 | 0.0% | 6.1% | 15.2% | 40.9% | 37.9% |
| 34 | Shoulder Dystocia – March 2012 | 25 | 0.0% | 4.0% | 4.0% | 40.0% | 52.0% |
| 35 | The Management of Women with Red Cell Antibodies during Pregnancy – May 2014 | 54 | 0.0% | 3.7% | 13.0% | 29.6% | 53.7% |
| 36 | Umbilical Cord Prolapse – November 2014 | 46 | 0.0% | 8.7% | 6.5% | 39.1% | 45.7% |
| 37 | Vasa Praevia Diagnosis and Management – September 2018 | 13 | 0.0% | 15.4% | 0.0% | 30.8% | 53.8% |

Table S5: Frequences of grading of recommendations in Obstetric Green top Guidelines

| **Guideline Name** | **Recommendation** | **Number of studies on which recommendation based** | **Pre MMR**  **vs**  **Post MMR** | **Score/median and range**  **for health equity score** | **Score/median and range for**  **generalisability** |
| --- | --- | --- | --- | --- | --- |
| Identification and management of maternal sepsis during and following pregnancy | Close household contacts of women with GAS infection should be warned to seek medical attention should symptoms develop and may warrant antibiotic prophylaxis. | 1 | Post-2004 | 4 | 0 |
| Care of late intrauterine fetal death and stillbirth | Women should be advised that almost one-third of those who choose non-pharmacological measures to suppress lactation experience excessive discomfort. | 1 | Pre-2004 | 0 | 8 |
| Care of late intrauterine fetal death and stillbirth | Women should be advised that dopamine agonists successfully suppress lactation and are generally well tolerated; cabergoline may be superior to bromocriptine. | 1 | Pre-2004 | 0 | 8 |
| Care of late intrauterine fetal death and stillbirth | The opportunity to spend time with a baby, and to make memories with a baby should be actively supported and offered. | 1 | Post-2004 | 0 | 1 |
| Care of late intrauterine fetal death and stillbirth | It is reasonable to offer parents a chance to see their baby more than once, and they should be informed that they can change their mind at any point, but once this decision has been made it should be respected. | 1 | Post-2004 | 0 | 1 |
| Care of late intrauterine fetal death and stillbirth | With regards to inter-pregnancy intervals, it is important to balance physical and psychological considerations. | 2 | 2 post-2004 | 3 (1-5) | 3.5 (3-4) |
| Care of late intrauterine fetal death and stillbirth | Healthcare professionals should be offered training and support when caring for parents who experience a late IUFD. | 1 | Post-2004 | 0 | 1 |
| Care of late intrauterine fetal death and stillbirth | The single most important risk factor for recurrent late IUFD is the history of previous late IUFD. A woman's recurrence risk should be stratified based on the investigations following index late IUFD and other known maternal risk factors. | 1 | Post-2004 | 1 | 6 |
| Care of late intrauterine fetal death and stillbirth | Women and families who have experienced prior late IUFD may need emotional support and should be provided with support during pregnancy. | 1 | Post-2004 | 1 | 2 |
| Amniocentesis and CVS | Amniocentesis should be performed after 15weeks gestation. | 1 | Pre-2004 | 1 | 5 |
| Cerclage | Women with a history of one or more spontaneous second trimester loss or preterm births who are undergoing ultrasound surveillance of cervical length should be offered cerclage if the cervix is 25mm or less at gestations less than 24 weeks | 1 | Post-2004 | 2 | 9 |
| Cerclage | In women with a previous unsuccessful transvaginal cerclage, insertion of a transabdominal cerclage may be discussed and considered | 1 | Post-2004 | 3 | 7 |
| Antepartum haemorrhage | Clinicians should offer a single course of antenatal corticosteroids to women between 24+0 and 34+6 weeks of gestation at risk of preterm birth. | 1 | Post-2004 | 0 | 0 |
| Antepartum haemorrhage | Women with APH resulting from placental abruption or placenta praevia should be strongly recommended to receive active management of the third stage of labour. | 1 | Post-2004 | 0 | 4 |
| Care of Women Presenting with Suspected Preterm Prelabour Rupture of Membranes from 24+0 Weeks of Gestation | Erythromycin should be given for 10 days following the diagnosis of PPROM, or until the woman is in established labour (whichever is sooner) | 1 | Post-2004 | 0 | 7 |
| Care of Women Presenting with Suspected Preterm Prelabour Rupture of Membranes from 24+0 Weeks of Gestation | In women who have PPROM from 24 +0 weeks, antenatal corticosteroids should be: offered between 26+0 and 33+6 weeks of gestation | 1 | Post-2004 | 0 | 0 |
| Care of Women Presenting with Suspected Preterm Prelabour Rupture of Membranes from 24+0 Weeks of Gestation | In women who have PPROM from 24+0 weeks, antenatal corticosteroids should be: considered between 34+0 and 35+6 weeks of gestation | 1 | Post-2004 | 0 | 0 |
| Care of Women Presenting with Suspected Preterm Prelabour Rupture of Membranes from 24+0 Weeks of Gestation | In women who have PPROM and are in established labour or having a planned preterm birth within 24 hours, intravenous magnesium sulfate should be offered between 24+0 and 29+6 weeks of gestation | 1 | Post-2004 | 0 | 7 |
| Care of Women Presenting with Suspected Preterm Prelabour Rupture of Membranes from 24+0 Weeks of Gestation | Tocolysis in patients with PPROM is not recommended | 1 | Post-2004 | 0 | 6 |
| Care of Women Presenting with Suspected Preterm Prelabour Rupture of Membranes from 24+0 Weeks of Gestation | Women whose pregnancy is complicated by PPROM after 24 +0 weeks’ gestation and who have no contraindications to continuing the pregnancy should be offered expectant management until 37 +0 weeks; timing of birth should be discussed with each woman on an individual basis with careful consideration of patient preference and ongoing clinical assessment | 1 | Post-2004 | 0 | 7 |
| The Management of Nausea and Vomiting in Pregnancy and Hyperemesis Gravidarum | Ketonuria is not an indicator of dehydration in pregnancy and should not be used to assess severity. | 1 | Post-2004 | 0 | 3 |
| The Management of Nausea and Vomiting in Pregnancy and Hyperemesis Gravidarum | Corticosteroids should be reserved for cases where standard therapies have been ineffective and used in combination with antiemetics. | 1 | Post-2004 | 0 | 6 |
| The Management of Nausea and Vomiting in Pregnancy and Hyperemesis Gravidarum | First line - There are safety data for antiemetics such as anti (H1) histamines, phenothiazines and pyridoxine-doxylamine (Xonvea®) and they should be prescribed initially when required for NVP and HG | 1 | Post-2004 | 0 | 8 |
| Intrahepatic cholestasis of pregnancy | Advise women with isolated ICP and a singleton pregnancy that the risk of stillbirth only increases above population rate once their serum bile acid concentration is 100 micromol/L or more: In women with peak bile acids 19–39 micromol/L and no other risk factors, advise them that the risk of stillbirth is similar to the background risk. In women with peak bile acids 40–99 micromol/L and no other risk factors, advise them that the risk of stillbirth is similar to the background risk until 38–39 weeks' gestation. In women with peak bile acids 100 micromol/L or more, advise them that the risk of stillbirth is higher than the background risk | 1 | Post-2004 | 3 | 6 |
| Intrahepatic cholestasis of pregnancy | Advise women with moderate or severe ICP that they have a higher chance of both spontaneous and iatrogenic preterm birth | 1 | Post-2004 | 3 | 6 |
| Intrahepatic cholestasis of pregnancy | Advise women with moderate or severe ICP that they have an increased chance of having meconium-stained amniotic fluid during labour and birth | 1 | Post-2004 | 3 | 6 |
| Intrahepatic cholestasis of pregnancy | Advise women with moderate or severe ICP that their baby is more likely to receive neonatal care | 1 | Post-2004 | 3 | 6 |
| Intrahepatic cholestasis of pregnancy | Advise women that there are no treatments that improve pregnancy outcome (or raised bile acid concentrations) and treatments to improve maternal itching are of limited benefit | 1 | Post-2004 | 0 | 8 |
| Intrahepatic cholestasis of pregnancy | Do not routinely offer ursodeoxycholic acid for the purpose of reducing adverse perinatal outcomes in women with ICP | 1 | Post-2004 | 4 | 9 |
| Intrahepatic cholestasis of pregnancy | Consider options of planned birth by 40 weeks' gestation or ongoing antenatal care according to national guidance in women with mild ICP (peak bile acids 19–39 micromol/L) and no other risk factors; advise women that the risk of stillbirth is similar to the background risk | 1 | Post-2004 | 3 | 6 |
| Intrahepatic cholestasis of pregnancy | Consider planned birth at 38–39 weeks' gestation in women with moderate ICP with peak bile acids 40–99 micromol/L and no other risk factors; advise them that the overall risk of stillbirth is similar to the background risk until 38–39 weeks' gestation | 1 | Post-2004 | 3 | 6 |
| Intrahepatic cholestasis of pregnancy | Consider planned birth at 35–36 weeks' gestation in women with severe ICP with peak bile acids 100 micromol/L or more; advise them that the risk of stillbirth is higher than the background risk | 1 | Post-2004 | 3 | 6 |
| Investigation and Care of a Small-for-Gestational-Age Fetus and a Growth Restricted Fetus | Women at risk of pre-eclampsia and/or placental dysfunction should take aspirin 150 mg once daily at night from 12+0–36+6 weeks of pregnancy to reduce their chance of SGA and FGR. | 1 | Post-2004 | 1 | 8 |
| Investigation and Care of a Small-for-Gestational-Age Fetus and a Growth Restricted Fetus | LMWH should not be prescribed to reduce the risk of SGA or FGR in at-risk women. | 7 | 7 post-2004 | 3 (0-4) | 7 (6-9) |
| Investigation and Care of a Small-for-Gestational-Age Fetus and a Growth Restricted Fetus | Routine measurement of fetal AC or EFW in the third trimester does not reduce the incidence of an SGA newborn nor does it improve perinatal outcome. | 4 | 4 post-2004 | 2 (0-3) | 6.5 (6-7) |
| Investigation and Care of a Small-for-Gestational-Age Fetus and a Growth Restricted Fetus | LMWH should not be prescribed to women in whom fetal growth disorders have been diagnosed. | 3 | 3 post-2004 | 3 (2-4) | 7 (7-8) |
| Investigation and Care of a Small-for-Gestational-Age Fetus and a Growth Restricted Fetus | Women should not be prescribed phosphodiesterase 5 (PDE5) inhibitors to treat FGR outside of RCTs. | 6 | 6 post-2004 | 1 (0-4) | 5.5 (4-9) |
| Investigation and Care of a Small-for-Gestational-Age Fetus and a Growth Restricted Fetus | In pregnancies with FGR, birth should be initiated from 37+0 weeks to be completed by 37+6 weeks. | 2 | 2 post-2004 | 1.5 (1-2) | 5 (2-8) |
| Investigation and Care of a Small-for-Gestational-Age Fetus and a Growth Restricted Fetus | Antenatal corticosteroids should be offered to women between 24 +0 and 34 +6weeks, ideally 48 hours before an anticipated birth. | 1 | Post-2004 | 0 | 0 |
| External Cephalic Version and Reducing the Incidence of Term Breech Presentation | Women should be informed that the success rate of ECV is approximately 50%. | 1 | Post-2004 | 0 | 6 |
| External Cephalic Version and Reducing the Incidence of Term Breech Presentation | Women should be informed that a successful ECV reduces the chance of caesarean section. | 1 | Post-2004 | 0 | 6 |
| External Cephalic Version and Reducing the Incidence of Term Breech Presentation | Use of tocolysis with betamimetics improves the success rates of ECV | 4 | 4 post-2004 | 1.5 (0-2) | 6.5 (5-8) |
| Management of Breech Presentation | Women with a breech presentation at term should be offered ECV unless there is an absolute contraindication. They should be advised on the risks and benefits of ECV and the implications for mode of delivery. | 1 | Post-2004 | 0 | 6 |
| Management of Breech Presentation | Women should be informed that planned caesarean section leads to a small reduction in perinatal mortality compared with planned vaginal breech delivery. Any decision to perform a caesarean section needs to be balanced against the potential adverse consequences that may result from this. | 2 | 2 post-2004 | 0.5 (0-1) | 6 (6,6) |
| Management of Breech Presentation | Women should be informed that planned caesarean section for breech presentation at term carries a small increase in immediate complications for the mother compared with planned vaginal birth. | 2 | 2 post-2004 | 1 (1,1) | 5.5 (5-6) |
| Reduced Fetal Movements | Ultrasound should include assessment of fetal morphology if this has not previously been performed and the woman has no objection to this being carried out. | 1 | Post-2004 | 0 | 2 |
| Maternal Collapse in Pregnancy and the Puerperium | Intravenous tranexamic acid significantly reduces mortality due to postpartum haemorrhage. | 1 | Post-2004 | 1 | 8 |
| Maternal Collapse in Pregnancy and the Puerperium | Life support training improves resuscitation skills. | 1 | Post-2004 | 0 | 2 |
| The Management of Third- and Fourth-Degree Perineal Tears | Warm compression during the second stage of labour reduces the risk of OASIS. | 1 | Post-2004 | 1 | 7 |
| The Management of Third- and Fourth-Degree Perineal Tears | For repair of a full thickness external anal sphincter (EAS) tear, either an overlapping or an end-to- end (approximation) method can be used with equivalent outcomes. | 1 | Post-2004 | 0 | 7 |
| Reducing the Risk of VTE during Pregnancy and the Puerperium | LMWHs are the agents of choice for antenatal and postnatal thromboprophylaxis. | 7 | 4 pre-2004 and 3 Post-2004 | 0 (0,0,0,0,0,0,0) | 3 (0-6) |
| Reducing the Risk of VTE during Pregnancy and the Puerperium | LMWH is safe in breastfeeding. | 1 | Post-2004 | 0 | 2 |
| Management of Beta Thalassaemia in Pregnancy | Folic acid (5 mg) is recommended preconceptually to all women to prevent neural tube defects. | 2 | 1 pre-2004 and 1 post-2004 | 0.5 (0-1) | 3 (0-6) |
| Management of Beta Thalassaemia in Pregnancy | Active management of the third stage of labour is recommended to minimise blood loss. | 2 | 2 post-2004 | 0 (0) | 3.5 (0-7) |
| Prevention and Mx of PPH | Uterine massage is of no benefit in the prophylaxis of PPH. | 1 | Post-2004 | 0 | 6 |
| Prevention and Mx of PPH | Prophylactic uterotonics should be routinely offered during the third stage of labour. | 4 | 4 post-2004 | 0 (0,0,0,0) | 5 (0-8) |
| Prevention and Mx of PPH | Oxytocin 10 iu IM is the agent of choice for PPH prophylaxis in vaginal delivery. | 1 | Post-2004 | 3 | 7 |
| Prevention and Mx of PPH | Consider IV tranexamic acid (0.5–1.0 g) with oxytocin in women at increased PPH risk at caesarean. | 1 | Post-2004 | 1 | 7 |
| Mx of Monochorionic Twin Pregnancy | cff DNA testing (NIPT) contingent on the first trimester combined test is recommended for twin pregnancies including monochorionic twins. | 2 | 2 post-2004 | 0.5 (0-1) | 3.5 (3-4) |
| Mx of Monochorionic Twin Pregnancy | TTTS before 26 weeks should be treated by fetoscopic laser ablation rather than amnioreduction or septostomy. | 7 | 7 post-2004 | 0 (0-2) | 6 (3-7) |
| Mx of Monochorionic Twin Pregnancy | Fetoscopic laser ablation should follow the serial, sequential SOLOMON technique. | 2 | 2 post-2004 | 0.5 (0-1) | 7 (7,7) |
| Mx of Monochorionic Twin Pregnancy | It is appropriate to aim for vaginal birth of MCDA twins unless there are other specific clinical indications. | 1 | Post-2004 | 1 | 3 |
| Malaria in Pregnancy and Prevention | Inform women about bite prevention measures including repellents, insecticide-treated bed nets, knock-down sprays, protective clothing, and room protection. | 3 | 2 pre-2004 and 1 post-2004 | 1 (1-2) | 5 (4-6) |
| Malaria in Pregnancy and Prevention | Inform women (and their GP) of the risks and benefits of chemoprophylaxis versus malaria. | 7 | 6 pre-2004 and 1 post-2004 | 2 (1-6) | 6 (4-8) |
| Malaria in Pregnancy and Prevention | Remind women that no malaria prophylaxis regimen is 100% protective. | 1 | Pre-2004 | 2 | 8 |
| [The Diagnosis and Treatment of Malaria in Pregnancy](https://www.rcog.org.uk/guidance/browse-all-guidance/green-top-guidelines/the-diagnosis-and-treatment-of-malaria-in-pregnancy-green-top-guideline-no-54b/) | Microscopic diagnosis allows species identification and estimation of parasitaemia, enabling appropriate treatment. | 2 | 2 post-2004 | 1 (0-2) | 3.5 (0-7) |
| [The Diagnosis and Treatment of Malaria in Pregnancy](https://www.rcog.org.uk/guidance/browse-all-guidance/green-top-guidelines/the-diagnosis-and-treatment-of-malaria-in-pregnancy-green-top-guideline-no-54b/) | Admit all pregnant women with uncomplicated malaria to hospital; severe cases to intensive care. | 1 | Post-2004 | 1 | 0 |
| [The Diagnosis and Treatment of Malaria in Pregnancy](https://www.rcog.org.uk/guidance/browse-all-guidance/green-top-guidelines/the-diagnosis-and-treatment-of-malaria-in-pregnancy-green-top-guideline-no-54b/) | Use IV artesunate for severe falciparum malaria; IV quinine if artesunate unavailable. | 1 | Post-2004 | 1 | 0 |
| [The Diagnosis and Treatment of Malaria in Pregnancy](https://www.rcog.org.uk/guidance/browse-all-guidance/green-top-guidelines/the-diagnosis-and-treatment-of-malaria-in-pregnancy-green-top-guideline-no-54b/) | Use chloroquine to treat P. vivax, P. ovale, and P. malariae. | 1 | Post-2004 | 1 | 0 |
| [The Diagnosis and Treatment of Malaria in Pregnancy](https://www.rcog.org.uk/guidance/browse-all-guidance/green-top-guidelines/the-diagnosis-and-treatment-of-malaria-in-pregnancy-green-top-guideline-no-54b/) | Screen and treat for anaemia in women with malaria. | 1 | Pre-2004 | 3 | 5 |
| [The Diagnosis and Treatment of Malaria in Pregnancy](https://www.rcog.org.uk/guidance/browse-all-guidance/green-top-guidelines/the-diagnosis-and-treatment-of-malaria-in-pregnancy-green-top-guideline-no-54b/) | Uncomplicated malaria in pregnancy is not an indication for induction of labour. | 6 | 5 pre-2004 and 1 post-2004 | 1.5 (0-4) | 5.5 (0-7) |
| [Management of Inherited Bleeding Disorders in Pregnancy](https://www.rcog.org.uk/guidance/browse-all-guidance/green-top-guidelines/management-of-inherited-bleeding-disorders-in-pregnancy-green-top-guideline-no-71/) | The incidence in the non-Jewish population is 1 in 1,000,000; higher in Ashkenazi Jews (heterozygosity 8–9%, homozygosity 0.2–0.5%). | 4 | 4 pre-2004 | 1 (0-2) | 1 (0-3) |
| [Management of Inherited Bleeding Disorders in Pregnancy](https://www.rcog.org.uk/guidance/browse-all-guidance/green-top-guidelines/management-of-inherited-bleeding-disorders-in-pregnancy-green-top-guideline-no-71/) | BSS is associated with high risk of primary and secondary PPH and wound haematoma. Delivery planning requires a multidisciplinary team. | 1 | Post-2004 | 2 | 2 |
| [Prevention of Early-onset Group B Streptococcal Disease](https://www.rcog.org.uk/guidance/browse-all-guidance/green-top-guidelines/prevention-of-early-onset-group-b-streptococcal-disease-green-top-guideline-no-36/) | In women with negative or unknown GBS status, offer induction of labour immediately or expectant management up to 24 hours. Beyond 24 hours, induction is appropriate. | 2 | 1 pre-2004 and 1 post-2004 | 1 (0-2) | 3 (0-6) |
| [Care of Women with Obesity in Pregnancy](https://www.rcog.org.uk/guidance/browse-all-guidance/green-top-guidelines/care-of-women-with-obesity-in-pregnancy-green-top-guideline-no-72/) | Active management of the third stage of labour should be recommended to reduce the risk of PPH. | 2 | 1 pre-2004 and 1 post-2004 | 1.5 (0-3) | 2 (0-4) |
| [Care of Women with Obesity in Pregnancy](https://www.rcog.org.uk/guidance/browse-all-guidance/green-top-guidelines/care-of-women-with-obesity-in-pregnancy-green-top-guideline-no-72/) | Women with class I obesity or greater having a caesarean section should receive prophylactic antibiotics at the time of surgery. | 1 | Post-2004 | 1 | 7 |
| [Care of Women with Obesity in Pregnancy](https://www.rcog.org.uk/guidance/browse-all-guidance/green-top-guidelines/care-of-women-with-obesity-in-pregnancy-green-top-guideline-no-72/) | Women undergoing caesarean section with >2 cm subcutaneous fat should have suturing of the tissue space to reduce wound infection and separation. | 3 | 2 pre-2004 and 1 post-2004 | 2 (0-3) | 0 (0-6) |
| Blood Transfusion in Obstetrics | Oral iron should be the preferred first-line treatment for iron deficiency. | 3 | 3 post-2004 | 0 (0,0,0) | 7 (6-8) |
| Blood Transfusion in Obstetrics | Active management of the third stage of labour should be recommended to reduce blood loss. | 3 | 3 pre-2004 | 1 (0-2) | 7 (1-8) |
| [Birth after Previous Caesarean Birth](https://www.rcog.org.uk/guidance/browse-all-guidance/green-top-guidelines/birth-after-previous-caesarean-birth-green-top-guideline-no-45/) | ERCS delivery should be conducted after 39+0 weeks of gestation. | 7 | 2 pre-2004 and 5 post-2004 | 1 (0-3) | 0 (0-9) |
| Assisted Vaginal Birth | Encourage women to have continuous support during labour to reduce the need for assisted vaginal birth. | 1 | Post-2004 | 1 | 7 |
| Assisted Vaginal Birth | Inform women that epidural analgesia may increase the need for assisted vaginal birth, though this is less likely with modern techniques. | 1 | Post-2004 | 0 | 4 |
| Assisted Vaginal Birth | Administering epidural in the latent phase does not increase assisted birth risk compared to active phase. | 1 | Post-2004 | 0 | 6 |
| Assisted Vaginal Birth | Encourage upright or lateral positions in second stage for women without epidurals. | 1 | Post-2004 | 0 | 5 |
| Assisted Vaginal Birth | Women with epidurals should adopt lying down lateral positions in second stage to increase spontaneous birth. | 2 | 2 post-2004 | 2 (0-4) | 8 (8,8) |
| Assisted Vaginal Birth | Do not discontinue epidural during pushing. | 1 | Post-2004 | 0 | 6 |
| Assisted Vaginal Birth | Do not recommend any specific regional technique to reduce assisted vaginal birth incidence. | 3 | 1 pre-2004 and 2 post-2004 | 0 (0-1) | 8 (5-8) |
| Assisted Vaginal Birth | Do not recommend routine oxytocin augmentation with epidural to reduce assisted birth. | 2 | 2 post-2004 | 0 (0,0) | 3 (0-6) |
| Assisted Vaginal Birth | Clinicians should be aware that ultrasound assessment of fetal head position prior to assisted vaginal birth is more reliable than clinical examination. | 2 | 2 post-2004 | 1.5 (1-2) | 6.5 (6-7) |
| Assisted Vaginal Birth | Forceps and vacuum each have distinct risks and benefits; vacuum is more likely to fail, forceps more likely to cause maternal trauma. | 2 | 2 pre-2004 | 0.5 (0-1) | 4 (3-5) |
| Assisted Vaginal Birth | Soft cup vacuum extractors have higher failure but lower neonatal scalp trauma than rigid cups. | 6 | 1 pre-2004 and 5 post-2004 | 1 (0-1) | 6 (5-6) |
| Assisted Vaginal Birth | A single prophylactic dose of intravenous amoxicillin and clavulanic acid should be given following assisted vaginal birth. | 3 | 3 post-2004 | 0 (0-3) | 8 (6-8) |
| Assisted Vaginal Birth | In the absence of contraindications, women should be offered regular NSAIDs and paracetamol after birth. | 1 | Post-2004 | 0 | 6 |
| [Antenatal corticosteroids to reduce neonatal morbidity and mortality](https://www.rcog.org.uk/guidance/browse-all-guidance/green-top-guidelines/antenatal-corticosteroids-to-reduce-neonatal-morbidity-and-mortality-green-top-guideline-no-74/) | A course of antenatal corticosteroids given within 7 days prior to preterm birth reduces perinatal and neonatal death and respiratory distress syndrome. | 1 | Post-2004 | 0 | 8 |
| [Antenatal corticosteroids to reduce neonatal morbidity and mortality](https://www.rcog.org.uk/guidance/browse-all-guidance/green-top-guidelines/antenatal-corticosteroids-to-reduce-neonatal-morbidity-and-mortality-green-top-guideline-no-74/) | Corticosteroids should be offered to women between 24+0 and 34+6 weeks’ gestation in whom imminent preterm birth is anticipated. | 2 | 2 post-2004 | 0.5 (0-1) | 4 (0-8) |
| [Antenatal corticosteroids to reduce neonatal morbidity and mortality](https://www.rcog.org.uk/guidance/browse-all-guidance/green-top-guidelines/antenatal-corticosteroids-to-reduce-neonatal-morbidity-and-mortality-green-top-guideline-no-74/) | Antenatal corticosteroids should be offered to women with PPROM who are at increased risk of preterm birth. | 1 | Post-2004 | 3 | 5 |
| Management of Thyroid Disorders in Pregnancy | Women on levothyroxine therapy for hypothyroidism should be counselled to self-initiate an empirical increase in their dose of levothyroxine by approximately 25%-30% as soon as they have a positive pregnancy test. This may be achieved by either: –doubling the dose of levothyroxine on two days of each week or– implementing a dose increment of: 25μg per day for women taking 100μg or less levothyroxine daily• 50μg per day for women taking greater than 100μglevothyroxine daily | 1 | Post-2004 | 1 | 5 |
| Management of Thyroid Disorders in Pregnancy | For pregnant women treated with levothyroxine for hypothyroidism, TSH and fT4concentrationsshould be checked every 4-6 weeks until 20 weeks of gestation then once again at 28 weeks of gestation | 1 | Post-2004 | 1 | 5 |
| Management of Thyroid Disorders in Pregnancy | Levothyroxine treatment is not recommended for women with TPOAb in the absence of thyroid dysfunction during pregnancy | 2 | 2 post-2004 | 3 (3,3) | 8 (8,8) |

Table S6: Median score for evidence underpinning each Grade A recommendations (recommendation Level). Ranges differ by level of aggregation (recommendation-level vs guideline-level medians).

| **Guideline Name** | **Recommendation** | **Evidence** | **Equity Domains Considered** | **Generalisability Domains Considered** |
| --- | --- | --- | --- | --- |
| Maternal Sepsis | Close household contacts of women with GAS infection should be warned to seek medical attention should symptoms develop, and may warrant antibiotic prophylaxis. | UK Health Security Agency, “UK Guidelines for the Managementof Contacts of Invasive Group A Streptococcus (iGAS) Infection inCommunity Settings,” https://assets.publishing.service.gov.uk/media/64071ec5d3bf7f25fa417a91/Management-of-contacts-of-invasive-group-a-streptococcus.pdf - guidance | AGE , VULNERABLE , RESIDENCE, OCCUPATION |  |
| Care of late intrauterine fetal death and stillbirth | Women should be advised that almost one-third of those who choose non-pharmacological measures to suppress lactation experience excessive discomfort. | Spitz AM, Lee NC, Peterson HB. Treatment for lactation suppression: little progress in one hundred years. Am J Obstet Gynecol.1998;179:1485–90. |  | RANDOMISATION, MASKING, ALLOCATION CONCEALMENT , MEASURES OF VARIABILITY , PRE SPECIFIED ANALYSIS , STATISTICAL METHODS , BASELINE DATA, ADDRESS MULTIPLICITY |
|  | Women should be advised that dopamine agonists successfully suppress lactation and are generally well tolerated; cabergoline may be superior to bromocriptine. | European Multicentre Study Group for Cabergoline in Lactation Inhibition. Single dose cabergoline versus bromocriptine in inhibi-tion of puerperal lactation: randomised, double blind, multicentrestudy. BMJ. 1991;302:1367–71. |  | RANDOMISATION, MASKING, ALLOCATION CONCEALMENT , MEASURES OF VARIABILITY , PRE SPECIFIED ANALYSIS , STATISTICAL METHODS , BASELINE DATA, ADDRESS MULTIPLICITY |
|  | The opportunity to spend time with a baby, and to make memories with a baby should be actively supported and offered. | Kingdon C, Givens JL, O’Donnell E, Turner M. Seeing and holdingbaby: systematic review of clinical management and parental out-comes after stillbirth. Birth. 2015;42:206. |  | PRE SPECIFIED ANALYSIS |
|  | It is reasonable to offer parents a chance to see their baby more than once, and they should be informed that they can change their mind at any point, but once this decision has been made it should be respected. | Kingdon C, Givens JL, O’Donnell E, Turner M. Seeing and holdingbaby: systematic review of clinical management and parental out-comes after stillbirth. Birth. 2015;42:206. sys rv |  | PRE SPECIFIED ANALYSIS |
|  | With regards to inter-pregnancy intervals, it is important to balance physical and psychological considerations. | risk but no absolute risk - Conde-Agudelo A, Rosas-Bermúdez A, Kafury-Goeta AC. Birthspacing and risk of adverse perinatal outcomes: a meta-analysis.JAMA. 2006;295:1809–23.207. | AGE , EDUCATION, SOCIOECONOMIC, RESIDENCE, OCCUPATION | MASKING, MEASURES OF VARIABILITY , PRE SPECIFIED ANALYSIS , STATISTICAL METHODS |
|  |  | risk (systematic rv) - Wendt A, Gibbs CM, Peters S, Hogue CJ. Impact of increasing inter-pregnancy interval on maternal and infant health. Paediatr PerinatEpidemiol. 2012;26(Suppl 1):239–58. | BMI | MEASURES OF VARIABILITY , PRE SPECIFIED ANALYSIS , STATISTICAL METHODS |
|  | Healthcare professionals should be offered training and support when caring for parents who experience a late IUFD. | Gandino G, Bernaudo A, Di Fini G, Vanni I, Veglia F. Healthcareprofessionals’ experiences of perinatal loss: a systematic review. JHealth Psychol. 2019;24:65–78. |  | PRE SPECIFIED ANALYSIS |
|  | The single most important risk factor for recurrent late IUFD is the history of previous late IUFD. A woman's recurrence risk should be stratified based on the investigations following index late IUFD and other known maternal risk factors. | Wojcieszek AM, Shepherd E, Middleton P, Lassi ZS, Wilson T,Murphy MM, et al. Care prior to and during subsequent preg-nancies following stillbirth for improving outcomes. CochraneDatabase Syst Rev. 2018c;12:CD012203 | SOCIOECONOMIC | MASKING, ALLOCATION CONCEALMENT , HANDLING OF WITHDRAWAL AND DROPOUTS , MEASURES OF VARIABILITY , PRE SPECIFIED ANALYSIS , STATISTICAL METHODS |
|  | Women and families who have experienced prior late IUFD may need emotional support and should be provided with support during pregnancy. | Mills TA, Ricklesford C, Cooke A, Heazell AE, Whitworth M,Lavender T. Parents’ experiences and expectations of care in preg-nancy after stillbirth or neonatal death: a metasynthesis. BJOG.2014;121:943–50 | EDUCATION | MEASURES OF VARIABILITY , PRE SPECIFIED ANALYSIS |
| Amniocentesis and CVS | Amniocentesis should be performed after 15weeks gestation. | Farrell SA, Summers AM, Dallaire L, Singer J, Johnson JA, Wilson RD.Club foot, an adverse outcome of early amniocentesis: disruption ordeformation? CEMAT. Canadian Early and Mid-TrimesterAmniocentesis Trial. J Med Genet 1999;36:843–6. | AGE | RANDOMISATION, MEASURES OF VARIABILITY , PRE SPECIFIED ANALYSIS , STATISTICAL METHODS , BASELINE DATA |
| Cerclage | Women with a history of one or more spontaneous second trimester loss or preterm births who are undergoing ultrasound surveillance of cervical length should be offered cerclage if the cervix is 25mm or less at gestations less than 24 weeks | Berghella V, Odibo AO, To MS, Rust OA, Althuisius SM. Cerclagefor short cervix on ultrasonography: meta-analysis of trials usingindividual patient-level data. Obstet Gynecol 2005;106:181–9 | AGE , RACE, ETHNICITY AND CULTURE | RANDOMISATION, MASKING, ALLOCATION CONCEALMENT , HANDLING OF WITHDRAWAL AND DROPOUTS , MEASURES OF VARIABILITY , PRE SPECIFIED ANALYSIS , STATISTICAL METHODS , BASELINE DATA, ADDRESS MULTIPLICITY |
|  | In women with a previous unsuccessful transvaginal cerclage, insertion of a transabdominal cerclage may be discussed and considered | Vousden NJ, Carter J, Seed PT, Shennan AH. What is the impactof preconception abdominal cerclage on fertility: evidence from a randomized controlled trial. Acta Obstet Gynecol Scand2017;96:543–6 | AGE , RACE, ETHNICITY AND CULTURE , BMI | RANDOMISATION, MASKING, MEASURES OF VARIABILITY , PRE SPECIFIED ANALYSIS , STATISTICAL METHODS , BASELINE DATA, ADDRESS MULTIPLICITY |
| Antepartum haemorrhage | Clinicians should offer a single course of antenatal corticosteroids to women between 24+0 and 34+6 weeks of gestation at risk of preterm birth. | Royal College of Obstetricians and Gynaecologists. Antenatal Corticosteroids to Reduce Neonatal Morbidity. Green-top Guideline No. 7. London: RCOG; 2010 |  |  |
|  | Women with APH resulting from placental abruption or placenta praevia should be strongly recommended to receive active management of the third stage of labour. | McDonald S, Abbott JM, Higgins SP. Prophylactic ergometrineoxytocin versus oxytocin for the third stage of labour. Cochrane Database Syst Rev 2004;(1):CD000201. |  | RANDOMISATION, HANDLING OF WITHDRAWAL AND DROPOUTS , PRE SPECIFIED ANALYSIS , STATISTICAL METHODS |
| Care of Women Presenting with Suspected Preterm Prelabour Rupture of Membranes from 24+0 Weeks of Gestation | Erythromycin should be given for 10 days following the diagnosis of PPROM, or until the woman is in established labour (whichever is sooner) | Kenyon S, Boulvain M, Neilson JP. Antibiotics for preterm rupture of membranes. Cochrane Database Syst Rev 2013;:CD001058 AND NG 25 |  | RANDOMISATION, MASKING, ALLOCATION CONCEALMENT , HANDLING OF WITHDRAWAL AND DROPOUTS , PRE SPECIFIED ANALYSIS , STATISTICAL METHODS , ADDRESS MULTIPLICITY |
|  | In women who have PPROM from 24 +0 weeks, antenatal corticosteroids should be: offered between 26+0 and 33+6 weeks of gestation | Magann EF, Haram K, Ounpraseuth S, Mortensen JH, Spencer HJ, Morrison JCMagann EF, Haram K, Ounpraseuth S, Mortensen JH, Spencer HJ, Morrison JC. Use of antenatal corticosteroids in special circumstances: a comprehensive review. Acta Obstet Gynecol Scand. 2017 Apr;96(4):395-409. doi: 10.1111/aogs.13104. PMID: 28130929.. Use of corticosteroids in special circumstances: a comprehensive review - metaanalysis. |  |  |
|  | In women who have PPROM from 24 +0 weeks, antenatal corticosteroids should be: considered between 34+0 and 35+6 weeks of gestation | Magann EF, Haram K, Ounpraseuth S, Mortensen JH, Spencer HJ, Morrison JC. Use of antenatal corticosteroids in special circumstances: a comprehensive review. Acta Obstet Gynecol Scand. 2017 Apr;96(4):395-409. doi: 10.1111/aogs.13104. PMID: 28130929. |  |  |
|  | In women who have PPROM and are in established labour or having a planned preterm birth within 24 hours, intravenous magnesium sulfate should be offered between 24+0 and 29+6 weeks of gestation | Costantine MM, Weiner SJ, Eunice Kennedy Shriver National Institute of Child Health and Human Development Maternal-Fetal Medicine Units Network. Effects of antenatal exposure to magnesium sulfate on neuroprotection and mortality in preterm infants: a meta-analysis. Obstet Gynecol 2009;114:354–64 |  | RANDOMISATION, MASKING, ALLOCATION CONCEALMENT , MEASURES OF VARIABILITY , PRE SPECIFIED ANALYSIS , STATISTICAL METHODS , ADDRESS MULTIPLICITY |
|  | Tocolysis in patients with PPROM is not recommended | Mackeen AD, Seibel-Seamon J, Muhammad J, Baxter JK, Berghella V. Tocolytics for preterm premature rupture of membranes. Cochrane Database Syst Rev 2014;:CD007062. |  | RANDOMISATION, MASKING, ALLOCATION CONCEALMENT , MEASURES OF VARIABILITY , PRE SPECIFIED ANALYSIS , STATISTICAL METHODS |
|  | Women whose pregnancy is complicated by PPROM after 24 +0 weeks’ gestation and who have no contraindications to continuing the pregnancy should be offered expectant management until 37 +0 weeks; timing of birth should be discussed with each woman on an individual basis with careful consideration of patient preference and ongoing clinical assessment | Bond DM, Middleton P, Levett KM, van der Ham DP, Crowther CA, Buchanan SL, et al. Planned early birth versus expectant management for women with preterm prelabour rupture of membranes prior to 37 weeks’ gestation for improving pregnancy outcome. Cochrane Database of Syst Rev 2017;: CD004735. |  | RANDOMISATION, MASKING, ALLOCATION CONCEALMENT , HANDLING OF WITHDRAWAL AND DROPOUTS , MEASURES OF VARIABILITY , PRE SPECIFIED ANALYSIS , STATISTICAL METHODS |
| The Management of Nausea and Vomiting in Pregnancy and Hyperemesis Gravidarum | Ketonuria is not an indicator of dehydration in pregnancy and should not be used to assess severity. | Niemeijer MN, Grooten IJ, Vos N, et al. Diagnostic markers for hy-peremesis gravidarum: a systematic review and metaanalysis. AmJ Obstet Gynecol. Aug 2014;211(2):150.e1-15. |  | MEASURES OF VARIABILITY , PRE SPECIFIED ANALYSIS , STATISTICAL METHODS |
|  | Corticosteroids should be reserved for cases where standard therapies have been ineffective and used in combination with antiemetics. | Grooten IJ, Vinke ME, Roseboom TJ, Painter RC. A SystematicReview and Meta-Analysis of the Utility of Corticosteroids in theTreatment of Hyperemesis Gravidarum. Nutr Metab Insights.2015;8(Suppl 1):23–32 |  | RANDOMISATION, MASKING, ALLOCATION CONCEALMENT , MEASURES OF VARIABILITY , PRE SPECIFIED ANALYSIS , STATISTICAL METHODS |
|  | First line - There are safety data for antiemetics such as anti (H1) histamines, phenothiazines and pyridoxine-doxylamine (Xonvea®) and they should be prescribed initially when required for NVP and HG | Matthews A, Dowswell T, Haas DM, Doyle M, O'Mathúna DP.Interventions for nausea and vomiting in early pregnancy. CochraneDatabase Syst Rev. Sep 2010;(9):CD007575 |  | RANDOMISATION, MASKING, ALLOCATION CONCEALMENT , HANDLING OF WITHDRAWAL AND DROPOUTS , MEASURES OF VARIABILITY , PRE SPECIFIED ANALYSIS , STATISTICAL METHODS , ADDRESS MULTIPLICITY |
| Intrahepatic cholestasis of pregnancy | Advise women with isolated ICP and a singleton pregnancy that the risk of stillbirth only increases above population rate once their serum bile acid concentration is 100 micromol/L or more: In women with peak bile acids 19–39 micromol/L and no other risk factors, advise them that the risk of stillbirth is similar to the background risk. In women with peak bile acids 40–99 micromol/L and no other risk factors, advise them that the risk of stillbirth is similar to the background risk until 38–39 weeks' gestation. In women with peak bile acids 100 micromol/L or more, advise them that the risk of stillbirth is higher than the background risk | Ovadia C, Seed PT, Sklavounos A, Geenes V, Di Ilio C, ChambersJ, et al. Association of adverse perinatal outcomes of intrahe-patic cholestasis of pregnancy with biochemical markers: resultsof aggregate and individual patient data meta-analyses. Lancet.2019;393(10174):899–909 | AGE , RACE, ETHNICITY AND CULTURE , BMI | RANDOMISATION, MEASURES OF VARIABILITY , PRE SPECIFIED ANALYSIS , STATISTICAL METHODS , BASELINE DATA, ADDRESS MULTIPLICITY |
|  | Advise women with moderate or severe ICP that they have a higher chance of both spontaneous and iatrogenic preterm birth | Ovadia C, Seed PT, Sklavounos A, Geenes V, Di Ilio C, ChambersJ, et al. Association of adverse perinatal outcomes of intrahe-patic cholestasis of pregnancy with biochemical markers: resultsof aggregate and individual patient data meta-analyses. Lancet.2019;393(10174):899–909 | AGE , RACE, ETHNICITY AND CULTURE , BMI | RANDOMISATION, MEASURES OF VARIABILITY , PRE SPECIFIED ANALYSIS , STATISTICAL METHODS , BASELINE DATA, ADDRESS MULTIPLICITY |
|  | Advise women with moderate or severe ICP that they have an increased chance of having meconium stained amniotic fluid during labour and birth | Ovadia C, Seed PT, Sklavounos A, Geenes V, Di Ilio C, ChambersJ, et al. Association of adverse perinatal outcomes of intrahe-patic cholestasis of pregnancy with biochemical markers: resultsof aggregate and individual patient data meta-analyses. Lancet.2019;393(10174):899–909 | AGE , RACE, ETHNICITY AND CULTURE , BMI | RANDOMISATION, MEASURES OF VARIABILITY , PRE SPECIFIED ANALYSIS , STATISTICAL METHODS , BASELINE DATA, ADDRESS MULTIPLICITY |
|  | Advise women with moderate or severe ICP that their baby is more likely to receive neonatal care | Ovadia C, Seed PT, Sklavounos A, Geenes V, Di Ilio C, ChambersJ, et al. Association of adverse perinatal outcomes of intrahe-patic cholestasis of pregnancy with biochemical markers: resultsof aggregate and individual patient data meta-analyses. Lancet.2019;393(10174):899–909 | AGE , RACE, ETHNICITY AND CULTURE , BMI | RANDOMISATION, MEASURES OF VARIABILITY , PRE SPECIFIED ANALYSIS , STATISTICAL METHODS , BASELINE DATA, ADDRESS MULTIPLICITY |
|  | Advise women that there are no treatments that improve pregnancy outcome (or raised bile acid concentrations) and treatments to improve maternal itching are of limited benefit | Ovadia C, Sajous J, Seed PT, Patel K, Williamson NJ, Attilakos G,et al. Ursodeoxycholic acid in intrahepatic cholestasis of pregnancy:a systematic review and individual participant data meta-analysis.Lancet Gastroenterol Hepatol. 2021;6(7):547–58 |  | RANDOMISATION, MASKING, HANDLING OF WITHDRAWAL AND DROPOUTS , MEASURES OF VARIABILITY , PRE SPECIFIED ANALYSIS , STATISTICAL METHODS , BASELINE DATA, ADDRESS MULTIPLICITY |
|  | Do not routinely offer ursodeoxycholic acid for the purpose of reducing adverse perinatal outcomes in women with ICP | Chappell LC, Bell JL, Smith A, Linsell L, Juszczak E, Dixon PH, et al.Ursodeoxycholic acid versus placebo in women with intrahepaticcholestasis of pregnancy (PITCHES): a randomised controlled trial.Lancet. 2019;394(10201):849– 60 | AGE , RACE, ETHNICITY AND CULTURE , SOCIOECONOMIC, BMI | RANDOMISATION, MASKING, ALLOCATION CONCEALMENT , HANDLING OF WITHDRAWAL AND DROPOUTS , MEASURES OF VARIABILITY , PRE SPECIFIED ANALYSIS , STATISTICAL METHODS , BASELINE DATA, ADDRESS MULTIPLICITY |
|  | Consider options of planned birth by 40 weeks' gestation or ongoing antenatal care according to national guidance in women with mild ICP (peak bile acids 19–39 micromol/L) and no other risk factors; advise women that the risk of stillbirth is similar to the background risk | Ovadia C, Seed PT, Sklavounos A, Geenes V, Di Ilio C, ChambersJ, et al. Association of adverse perinatal outcomes of intrahe-patic cholestasis of pregnancy with biochemical markers: resultsof aggregate and individual patient data meta-analyses. Lancet.2019;393(10174):899–909 | AGE , RACE, ETHNICITY AND CULTURE , BMI | RANDOMISATION, MEASURES OF VARIABILITY , PRE SPECIFIED ANALYSIS , STATISTICAL METHODS , BASELINE DATA, ADDRESS MULTIPLICITY |
|  | Consider planned birth at 38–39 weeks' gestation in women with moderate ICP with peak bile acids 40–99 micromol/L and no other risk factors; advise them that the overall risk of stillbirth is similar to the background risk until 38–39 weeks' gestation | Ovadia C, Seed PT, Sklavounos A, Geenes V, Di Ilio C, ChambersJ, et al. Association of adverse perinatal outcomes of intrahe-patic cholestasis of pregnancy with biochemical markers: resultsof aggregate and individual patient data meta-analyses. Lancet.2019;393(10174):899–909 | AGE , RACE, ETHNICITY AND CULTURE , BMI | RANDOMISATION, MEASURES OF VARIABILITY , PRE SPECIFIED ANALYSIS , STATISTICAL METHODS , BASELINE DATA, ADDRESS MULTIPLICITY |
|  | Consider planned birth at 35–36 weeks' gestation in women with severe ICP with peak bile acids 100 micromol/L or more; advise them that the risk of stillbirth is higher than the background risk | Ovadia C, Seed PT, Sklavounos A, Geenes V, Di Ilio C, ChambersJ, et al. Association of adverse perinatal outcomes of intrahe-patic cholestasis of pregnancy with biochemical markers: resultsof aggregate and individual patient data meta-analyses. Lancet.2019;393(10174):899–909 | AGE , RACE, ETHNICITY AND CULTURE , BMI | RANDOMISATION, MEASURES OF VARIABILITY , PRE SPECIFIED ANALYSIS , STATISTICAL METHODS , BASELINE DATA, ADDRESS MULTIPLICITY |
| Investigation and Care of a Small-for-Gestational-Age Fetus and a Growth Restricted Fetus | Women at risk of pre-eclampsia and/or placental dysfunction should take aspirin 150 mg once daily at night from 12+0–36+6 weeks of pregnancy to reduce their chance of SGA and FGR. | Rolnik DL, Wright D, Poon LC, et al. Aspirin versus Placebo inPregnancies at High Risk for Preterm Preeclampsia. N Engl J Med.08 17 2017;377(7):613–22. https://doi.org/10.1056/ NEJMoa1704559 - multicenter, double-blind, placebo-controlled trial | AGE | RANDOMISATION, MASKING, ALLOCATION CONCEALMENT , HANDLING OF WITHDRAWAL AND DROPOUTS , PRE SPECIFIED ANALYSIS , STATISTICAL METHODS , BASELINE DATA, ADDRESS MULTIPLICITY |
|  | LMWH should not be prescribed to reduce the risk of SGA or FGR in at-risk women. | Dodd JM, McLeod A, Windrim RC, Kingdom J. Antithrombotictherapy for improving maternal or infant health outcomes in womenconsidered at risk of placental dysfunction. Cochrane Database SystRev Jul 24 2013;(7):CD006780. https://doi.org/10.1002/14651858.CD006780.pub3157. |  | RANDOMISATION, MASKING, ALLOCATION CONCEALMENT , HANDLING OF WITHDRAWAL AND DROPOUTS , MEASURES OF VARIABILITY , PRE SPECIFIED ANALYSIS , STATISTICAL METHODS , BASELINE DATA, ADDRESS MULTIPLICITY |
|  |  | Mastrolia SA, Novack L, Thachil J, et al. LMWH in the prevention of preeclampsia and fetal growth restriction in women withoutthrombophilia. A systematic review and meta-analysis. ThrombHaemost Oct 28 2016;116(5):868–78. https://doi.org/10.1160/TH16- 02- 0169158. |  | RANDOMISATION, MASKING, MEASURES OF VARIABILITY , PRE SPECIFIED ANALYSIS , STATISTICAL METHODS , BASELINE DATA |
|  |  | Haddad B, Winer N, Chitrit Y, et al. Enoxaparin and AspirinCompared With Aspirin Alone to Prevent Placenta-MediatedPregnancy Complications: A Randomized Controlled Trial. ObstetGynecol. 11 2016;128(5):1053–63. https://doi.org/10.1097/AOG.00000 00000 001673159. | AGE , RACE, ETHNICITY AND CULTURE , DISABILITY - (PHYSICAL,ITELLECTUAL,MENTAL, SUBSTANCE MISUSE), BMI | RANDOMISATION, ALLOCATION CONCEALMENT , HANDLING OF WITHDRAWAL AND DROPOUTS , MEASURES OF VARIABILITY , PRE SPECIFIED ANALYSIS , STATISTICAL METHODS , BASELINE DATA, ADDRESS MULTIPLICITY |
|  |  | Groom KM, McCowan LM, Mackay LK, et al. Enoxaparin for theprevention of preeclampsia and intrauterine growth restriction inwomen with a history: a randomized trial. Am J Obstet GynecolMar 2017;216(3):296.e1-296.e14. https://doi.org/10.1016/j.ajog.2017.01.014. | AGE , RACE, ETHNICITY AND CULTURE , BMI | RANDOMISATION, HANDLING OF WITHDRAWAL AND DROPOUTS , MEASURES OF VARIABILITY , PRE SPECIFIED ANALYSIS , STOPPING RULES , STATISTICAL METHODS , BASELINE DATA |
|  |  | Martinelli I, Ruggenenti P, Cetin I, et al. Heparin in pregnantwomen with previous placenta-mediated pregnancy complica-tions: a prospective, randomized, multicenter, controlled clinicaltrial. Blood. Apr 05 2012;119(14):3269–75. https://doi.org/10.1182/blood-2011-11-391383161. | AGE , RACE, ETHNICITY AND CULTURE , BMI | RANDOMISATION, ALLOCATION CONCEALMENT , HANDLING OF WITHDRAWAL AND DROPOUTS , MEASURES OF VARIABILITY , PRE SPECIFIED ANALYSIS , STOPPING RULES , STATISTICAL METHODS , BASELINE DATA, ADDRESS MULTIPLICITY |
|  |  | Abheiden C, Van Hoorn ME, Hague WM, Kostense PJ, van PampusMG, de Vries J. Does low-molecular-weight heparin influence fetalgrowth or uterine and umbilical arterial Doppler in women with ahistory of early-onset uteroplacental insufficiency and an inherit-able thrombophilia? Secondary randomised controlled trial resultsBJOG Apr 2016;123(5):797–805. https://doi.org/10.1111/1471- 0528.13421162. | AGE , RACE, ETHNICITY AND CULTURE , BMI | RANDOMISATION, ALLOCATION CONCEALMENT , HANDLING OF WITHDRAWAL AND DROPOUTS , MEASURES OF VARIABILITY , PRE SPECIFIED ANALYSIS , STATISTICAL METHODS , BASELINE DATA |
|  |  | Rodger MA, Gris JC, de Vries JIP, et al. Low-molecular-weight hep-arin and recurrent placenta-mediated pregnancy complications:a meta-analysis of individual patient data from randomised con-trolled trials. Lancet. 11 26 2016;388(10060):2629–4 | AGE , RACE, ETHNICITY AND CULTURE , BMI | RANDOMISATION, MASKING, MEASURES OF VARIABILITY , PRE SPECIFIED ANALYSIS , STATISTICAL METHODS , BASELINE DATA, ADDRESS MULTIPLICITY |
|  | Routine measurement of fetal AC or EFW in the third trimester does not reduce the incidence of an SGA newborn nor does it improve perinatal outcome. | Bricker L, Medley N, Pratt JJ. Routine ultrasound in late pregnancy(after 24 weeks' gestation). Cochrane Database Syst Rev. Jun 292015;(6):CD001451. |  | RANDOMISATION, MASKING, ALLOCATION CONCEALMENT , HANDLING OF WITHDRAWAL AND DROPOUTS , MEASURES OF VARIABILITY , PRE SPECIFIED ANALYSIS , STATISTICAL METHODS |
|  |  | GC S, AA M, D W et al. Universal late pregnancy ultrasound screen-ing to predict adverse outcomes in nulliparous women: a system-atic review and cost-effectiveness analysis. Health Technol Assess.2021;25(15):1–190. | AGE , EDUCATION, BMI | RANDOMISATION, MASKING, MEASURES OF VARIABILITY , PRE SPECIFIED ANALYSIS , STATISTICAL METHODS , ADDRESS MULTIPLICITY |
|  |  | Wanyonyi SZ, Orwa J, Ozelle H, et al. Routine third-trimester ul-trasound for the detection of small-for-gestational age in low-risk pregnancies (ROTTUS study): randomized controlled trial.Ultrasound Obstet Gynecol Jun 2021;57(6):910–6. | AGE , BMI | RANDOMISATION, HANDLING OF WITHDRAWAL AND DROPOUTS , MEASURES OF VARIABILITY , PRE SPECIFIED ANALYSIS , STATISTICAL METHODS , BASELINE DATA |
|  |  | Caradeux J, Martinez-Portilla RJ, Peguero A, Sotiriadis A, FiguerasF. Diagnostic performance of third-trimester ultrasound for theprediction of late-onset fetal growth restriction: a systematic reviewand meta-analysis. Am J Obstet Gynecol May 2019;220(5):449-459.e19 | AGE , BMI | RANDOMISATION, MASKING, HANDLING OF WITHDRAWAL AND DROPOUTS , MEASURES OF VARIABILITY , PRE SPECIFIED ANALYSIS , STATISTICAL METHODS , BASELINE DATA |
|  | LMWH should not be prescribed to women in whom fetal growth disorders have been diagnosed. | Haddad B, Winer N, Chitrit Y, et al. Enoxaparin and AspirinCompared With Aspirin Alone to Prevent Placenta-MediatedPregnancy Complications: A Randomized Controlled Trial. ObstetGynecol. 11 2016;128(5):1053–63. https://doi.org/10.1097/AOG.00000 00000 001673159. | AGE , RACE, ETHNICITY AND CULTURE , DISABILITY - (PHYSICAL,ITELLECTUAL,MENTAL, SUBSTANCE MISUSE), BMI | RANDOMISATION, ALLOCATION CONCEALMENT , HANDLING OF WITHDRAWAL AND DROPOUTS , MEASURES OF VARIABILITY , PRE SPECIFIED ANALYSIS , STATISTICAL METHODS , BASELINE DATA, ADDRESS MULTIPLICITY |
|  |  | Groom KM, McCowan LM, Mackay LK, et al. Enoxaparin for theprevention of preeclampsia and intrauterine growth restriction inwomen with a history: a randomized trial. Am J Obstet GynecolMar 2017;216(3):296.e1-296.e14. | AGE , RACE, ETHNICITY AND CULTURE , BMI | RANDOMISATION, HANDLING OF WITHDRAWAL AND DROPOUTS , MEASURES OF VARIABILITY , PRE SPECIFIED ANALYSIS , STOPPING RULES , STATISTICAL METHODS , BASELINE DATA |
|  |  | Abheiden C, Van Hoorn ME, Hague WM, Kostense PJ, van PampusMG, de Vries J. Does low-molecular-weight heparin influence fetalgrowth or uterine and umbilical arterial Doppler in women with ahistory of early-onset uteroplacental insufficiency and an inherit-able thrombophilia? Secondary randomised controlled trial resultsBJOG Apr 2016;123(5):797–805. https://doi.org/10.1111/1471- 0528.13421162. | AGE , RACE, ETHNICITY AND CULTURE , BMI | RANDOMISATION, ALLOCATION CONCEALMENT , HANDLING OF WITHDRAWAL AND DROPOUTS , MEASURES OF VARIABILITY , PRE SPECIFIED ANALYSIS , STATISTICAL METHODS , BASELINE DATA |
|  |  | Rodger MA, Gris JC, de Vries JIP, et al. Low-molecular-weight hep-arin and recurrent placenta-mediated pregnancy complications:a meta-analysis of individual patient data from randomised con-trolled trials. Lancet. 11 26 2016;388(10060):2629–41. | AGE , RACE, ETHNICITY AND CULTURE , BMI | RANDOMISATION, MASKING, MEASURES OF VARIABILITY , PRE SPECIFIED ANALYSIS , STATISTICAL METHODS , BASELINE DATA, ADDRESS MULTIPLICITY |
|  |  | Mazarico E, Molinet-Coll C, Martinez-Portilla RJ, Figueras F.Heparin therapy in placental insufficiency: Systematic review andmeta-analysis. Acta Obstet Gynecol Scand. 02 2020;99(2):167–74 | AGE , BMI | RANDOMISATION, ALLOCATION CONCEALMENT , HANDLING OF WITHDRAWAL AND DROPOUTS , MEASURES OF VARIABILITY , PRE SPECIFIED ANALYSIS , STATISTICAL METHODS , BASELINE DATA |
|  | Women should not be prescribed phosphodiesterase 5 (PDE5) inhibitors to treat FGR outside of RCTs. | Paauw ND, Terstappen F, Ganzevoort W, Joles JA, Gremmels H,Lely AT. Sildenafil During Pregnancy: A Preclinical Meta-Analysis on Fetal Growth and Maternal Blood Pressure. Hypertension.11 2017;70(5):998–1006. |  | HANDLING OF WITHDRAWAL AND DROPOUTS , MEASURES OF VARIABILITY , PRE SPECIFIED ANALYSIS , STATISTICAL METHODS , BASELINE DATA |
|  |  | von Dadelszen P, Dwinnell S, Magee LA, et al. for the Research into Advanced Fetal Diagnosis and Therapy (RAFT) Group. Sildenafilcitrate therapy for severe early- onset intrauterine growth restric-tion. BJOG Apr 2011;118(5):624–8. | AGE | MEASURES OF VARIABILITY , PRE SPECIFIED ANALYSIS , STATISTICAL METHODS , BASELINE DATA |
|  |  | Trapani A, Gonçalves LF, Trapani TF, Vieira S, Pires M, Pires MMS.Perinatal and Hemodynamic Evaluation of Sildenafil Citrate forPreeclampsia Treatment: A Randomized Controlled Trial. ObstetGynecol. 08 2016;128(2):253–9. |  | RANDOMISATION, MASKING, ALLOCATION CONCEALMENT , HANDLING OF WITHDRAWAL AND DROPOUTS , PRE SPECIFIED ANALYSIS , STATISTICAL METHODS |
|  |  | Sharp A, Cornforth C, Jackson R, et al. Maternal sildenafil forsevere fetal growth restriction (STRIDER): a multicentre, randomised, placebo-controlled, double-blind trial. Lancet ChildAdolesc Health. 02 2018;2(2):93–102. https://doi.org/10.1016/S2352-4642(17)30173- 6306. | AGE , RACE, ETHNICITY AND CULTURE , DISABILITY - (PHYSICAL,ITELLECTUAL,MENTAL, SUBSTANCE MISUSE), BMI | RANDOMISATION, MASKING, HANDLING OF WITHDRAWAL AND DROPOUTS , MEASURES OF VARIABILITY , PRE SPECIFIED ANALYSIS , STOPPING RULES , STATISTICAL METHODS , BASELINE DATA, ADDRESS MULTIPLICITY |
|  |  | Groom KM, McCowan LM, Mackay LK, et al. STRIDER NZAus: a multicentre randomised controlled trial of sildenafil therapy in early onset fetal growth restriction. BJOG 07 2019;126(8):997–1006. | AGE , RACE, ETHNICITY AND CULTURE , BMI | RANDOMISATION, MASKING, ALLOCATION CONCEALMENT , MEASURES OF VARIABILITY , PRE SPECIFIED ANALYSIS , STATISTICAL METHODS , BASELINE DATA, ADDRESS MULTIPLICITY |
|  |  | Kubo M, Tanaka H, Maki S, et al. Safety and dose-finding trial oftadalafil administered for fetal growth restriction: A phase-1 clinical study. J Obstet Gynaecol Res Jul 2017;43(7):1159–68 | AGE | MEASURES OF VARIABILITY , PRE SPECIFIED ANALYSIS , STOPPING RULES , STATISTICAL METHODS , BASELINE DATA |
|  | In pregnancies with FGR, birth should be initiated from 37+0 weeks to be completed by 37+6 weeks. | Thornton JG, Hornbuckle J, Vail A, Spiegelhalter DJ, Levene M,group Gs. Infant wellbeing at 2 years of age in the Growth Restriction Intervention Trial (GRIT): multicentred randomised controlledtrial. Lancet 2004 Aug 7–13 2004;364(9433):513–20. https://doi.org/10.1016/S0140- 6736(04)16809-8354. | AGE | RANDOMISATION, MASKING, ALLOCATION CONCEALMENT , MEASURES OF VARIABILITY , PRE SPECIFIED ANALYSIS , STATISTICAL METHODS , BASELINE DATA, ADDRESS MULTIPLICITY |
|  |  | National Institute for Health and Care Excellence, Preterm labourand birth. NICE Guideline [NG25]. London: NICE; 2015 (updated2019) | RACE, ETHNICITY AND CULTURE , SOCIOECONOMIC |  |
|  | Antenatal corticosteroids should be offered to women between 24+0 and 34+6 weeks, ideally 48 hours before an anticipated birth. | Stock SJ, Thomson AJ, Papworth S. on behalf of the Royal Collegeof Obstetricians and Gynaecologists. Antenatal corticosteroids toreduce neonatal morbidity and mortality: Green-top Guideline No.74. BJOG. Jul 2022;129(8):e35–e60. https://doi.org/10.1111/1471-0528.17027357. |  |  |
| External Cephalic Version and Reducing the Incidence of Term Breech Presentation | Women should be informed that the success rate of ECV is approximately 50%. | Hofmeyr GJ, Kulier R, West HM. External cephalic version forbreech presentation at term. Cochrane Database Syst Rev 2015;(4):CD000083 |  | RANDOMISATION, ALLOCATION CONCEALMENT , HANDLING OF WITHDRAWAL AND DROPOUTS , PRE SPECIFIED ANALYSIS , STATISTICAL METHODS , ADDRESS MULTIPLICITY |
|  | Women should be informed that a successful ECV reduces the chance of caesarean section. | Hofmeyr GJ, Kulier R, West HM. External cephalic version forbreech presentation at term. Cochrane Database Syst Rev 2015;(4):CD000083 |  | RANDOMISATION, ALLOCATION CONCEALMENT , HANDLING OF WITHDRAWAL AND DROPOUTS , PRE SPECIFIED ANALYSIS , STATISTICAL METHODS , ADDRESS MULTIPLICITY |
|  | Use of tocolysis with betamimetics improves the success rates of ECV | Cluver C, Gyte GM, Sinclair M, Dowswell T, Hofmeyr GJ.Interventions for helping to turn term breech babies to head ﬁrstpresentation when using external cephalic version. CochraneDatabase Syst Rev 2015;(2):CD000184.19. | AGE | RANDOMISATION, MASKING, ALLOCATION CONCEALMENT , HANDLING OF WITHDRAWAL AND DROPOUTS , PRE SPECIFIED ANALYSIS , STATISTICAL METHODS , BASELINE DATA, ADDRESS MULTIPLICITY |
|  |  | Wilcox CB, Nassar N, Roberts CL. Effectiveness of nifedipinetocolysis to facilitate external cephalic version: a systematicreview. BJOG 2011;118:423–8.20. |  | RANDOMISATION, MASKING, ALLOCATION CONCEALMENT , PRE SPECIFIED ANALYSIS , STATISTICAL METHODS |
|  |  | El-Sayed YY, Pullen K, Riley ET, Lyell D, Druzin ML, Cohen SE,et al. Randomized comparison of intravenous nitroglycerin andsubcutaneous terbutaline for external cephalic version undertocolysis. Am J Obstet Gynecol 2004;191:2051–5.21. | AGE , BMI | RANDOMISATION, ALLOCATION CONCEALMENT , MEASURES OF VARIABILITY , PRE SPECIFIED ANALYSIS , STATISTICAL METHODS , BASELINE DATA |
|  |  | Impey L, Pandit M. Tocolysis for repeat external cephalic versionin breech presentation at term: a randomised, double-blinded,placebo-controlled trial. BJOG 2005;112:627–31 | AGE , RACE, ETHNICITY AND CULTURE | RANDOMISATION, MASKING, ALLOCATION CONCEALMENT , MEASURES OF VARIABILITY , PRE SPECIFIED ANALYSIS , STATISTICAL METHODS , BASELINE DATA |
| Management of Breech Presentation | Women with a breech presentation at term should be offered ECV unless there is an absolute contraindication. They should be advised on the risks and benefits of ECV and the implications for mode of delivery. | ?Hofmeyr GJ, Kulier R, West HM. External cephalic version forbreech presentation at term. Cochrane Database Syst Rev 2015;(4):CD000083 |  | RANDOMISATION, ALLOCATION CONCEALMENT , HANDLING OF WITHDRAWAL AND DROPOUTS , PRE SPECIFIED ANALYSIS , STATISTICAL METHODS , ADDRESS MULTIPLICITY |
|  | Women should be informed that planned caesarean section leads to a small reduction in perinatal mortality compared with planned vaginal breech delivery. Any decision to perform a caesarean section needs to be balanced against the potential adverse consequences that may result from this. | Hofmeyr GJ, Hannah M, Lawrie TA. Planned caesarean section forterm breech delivery. Cochrane Database Syst Rev 2015;(7):CD000166.8. |  | RANDOMISATION, ALLOCATION CONCEALMENT , HANDLING OF WITHDRAWAL AND DROPOUTS , PRE SPECIFIED ANALYSIS , STATISTICAL METHODS , ADDRESS MULTIPLICITY |
|  |  | Whyte H, Hannah ME, Saigal S, Hannah WJ, Hewson S,Amankwah K, et al.; Term Breech Trial Collaborative Group.Outcomes of children at 2 years after planned cesarean birthversus planned vaginal birth for breech presentation at term: theInternational Randomized Term Breech Trial. Am J Obstet Gynecol2004;191:864–71 | AGE | RANDOMISATION, MASKING, ALLOCATION CONCEALMENT , PRE SPECIFIED ANALYSIS , STATISTICAL METHODS , BASELINE DATA |
|  | Women should be informed that planned caesarean section for breech presentation at term carries a small increase in immediate complications for the mother compared with planned vaginal birth. | Hofmeyr GJ, Hannah M, Lawrie TA. Planned caesarean section forterm breech delivery. Cochrane Database Syst Rev 2015;(7):CD000166. | SEXUAL ORIENTATION | RANDOMISATION, ALLOCATION CONCEALMENT , HANDLING OF WITHDRAWAL AND DROPOUTS , PRE SPECIFIED ANALYSIS , STATISTICAL METHODS , ADDRESS MULTIPLICITY |
|  |  | Hannah ME, Whyte H, Hannah WJ, Hewson S, Amankwah K,Cheng M, et al.; Term Breech Trial Collaborative Group.Maternal outcomes at 2 years after planned cesarean section versus planned vaginal birth for breech presentation at term: theinternational randomized Term Breech Trial. Am J Obstet Gynecol2004;191:917–27 | AGE | RANDOMISATION, ALLOCATION CONCEALMENT , PRE SPECIFIED ANALYSIS , STATISTICAL METHODS , BASELINE DATA |
| Reduced Fetal Movements | Ultrasound should include assessment of fetal morphology if this has not previously been performed and the woman has no objection to this being carried out. | Frøen JF,Tveit JV, Saastad E, Børdahl PE, Stray-Pedersen B, Heazell AE, et al. Management of decreased fetal movements. Semin Perinatol 2008;32:307–11-Cohort study |  | PRE SPECIFIED ANALYSIS , STOPPING RULES |
| Maternal Collapse in Pregnancy and the Puerperium | Intravenous tranexamic acid significantly reduces mortality due to postpartum haemorrhage. | WOMAN Trial Collaborators. Effect of early tranexamic acidadministration on mortality, hysterectomy, and other morbiditiesin women with post-partum haemorrhage (WOMAN): aninternational, randomised, double-blind, placebo-controlled trial.Lancet 2017;389:2105–16 | AGE | RANDOMISATION, MASKING, ALLOCATION CONCEALMENT , HANDLING OF WITHDRAWAL AND DROPOUTS , PRE SPECIFIED ANALYSIS , STOPPING RULES , STATISTICAL METHODS , ADDRESS MULTIPLICITY |
|  | Life support training improves resuscitation skills. | Merien AE, van de Ven J, Mol BW, Houterman S, Oei SG.Multidisciplinary team training in a simulation setting for acute obstetric emergencies: a systematic review. Obstet Gynecol 2010May;115:1021–31. |  | RANDOMISATION, PRE SPECIFIED ANALYSIS |
| The Management of Third- and Fourth-Degree Perineal Tears | Warm compression during the second stage of labour reduces the risk of OASIS. | Aasheim V, Nilsen AB, Lukasse M, Reinar LM. Perineal techniques during the second stage of labour for reducing perineal trauma. Cochrane Database Syst Rev 2011;(12):CD006672. | RACE, ETHNICITY AND CULTURE | RANDOMISATION, MASKING, ALLOCATION CONCEALMENT , HANDLING OF WITHDRAWAL AND DROPOUTS , PRE SPECIFIED ANALYSIS , STATISTICAL METHODS , BASELINE DATA |
|  | For repair of a full thickness external anal sphincter (EAS) tear, either an overlapping or an end-to- end (approximation) method can be used with equivalent outcomes. | Fernando RJ, Sultan AH, Kettle C, Thakar R. Methods of repair for obstetric anal sphincter injury. Cochrane Database Syst Rev 2013;(12):CD002866. |  | RANDOMISATION, MASKING, ALLOCATION CONCEALMENT , HANDLING OF WITHDRAWAL AND DROPOUTS , MEASURES OF VARIABILITY , PRE SPECIFIED ANALYSIS , STATISTICAL METHODS |
| Reducing the Risk of VTE during Pregnancy and the Puerperium | LMWHs are the agents of choice for antenatal and postnatal thromboprophylaxis. | Greer IA, Nelson-Piercy C. Low-molecular-weight heparins for thromboprophylaxis and treatment of venous thromboembolism in pregnancy: a systematic review of safety and efficacy. Blood 2005;106:401–7 |  | PRE SPECIFIED ANALYSIS , STATISTICAL METHODS |
|  |  | Sanson BJ, Lensing AW, Prins MH, Ginsberg JS, Barkagan ZS,Lavenne-Pardonge E, et al. Safety of low-molecular-weight heparin in pregnancy: a systematic review. Thromb Haemost 1999;81:668–72. |  | PRE SPECIFIED ANALYSIS , STATISTICAL METHODS , ADDRESS MULTIPLICITY |
|  |  | Ensom MH, Stephenson MD. Low-molecular-weight heparins in pregnancy. Pharmacotherapy 1999;19:1013–25.-systematic. |  | RANDOMISATION, MEASURES OF VARIABILITY , PRE SPECIFIED ANALYSIS |
|  |  | Warkentin TE, Levine MN, Hirsh J, Horsewood P, Roberts RS, Gent M, et al. Heparin-induced thrombocytopenia in patients treated with low-molecular-weight heparin or unfractionated heparin. N Engl J Med 1995;332:1330–5 - rct. |  | RANDOMISATION, MASKING, PRE SPECIFIED ANALYSIS , STATISTICAL METHODS |
|  |  | Warkentin TE, Greinacher A, Koster A, Lincoff AM; American College of Chest Physicians. Treatment and prevention of heparin-induced thrombocytopenia: American College of Chest Physicians Evidence-Based Clinical Practice Guidelines (8th Edition). Chest 2008;133 6 Suppl:340S–380S. |  |  |
|  |  | Pettilä V, Leinonen P, Markkola A, Hiilesmaa V, Kaaja R. Postpartum bone mineral density in women treated for thromboprophylaxis with unfractionated heparin or LMW heparin. Thromb Haemost 2002;87:182–6.-rct. |  |  |
|  |  | Tooher R, Gates S, Dowswell T, Davis LJ. Prophylaxis for venous thromboembolic disease in pregnancy and the early postnatal period. Cochrane Database Syst Rev 2010;(5):CD001689.m. Green-top Guideline No.37b |  | RANDOMISATION, MASKING, ALLOCATION CONCEALMENT , HANDLING OF WITHDRAWAL AND DROPOUTS , PRE SPECIFIED ANALYSIS , STATISTICAL METHODS |
|  | LMWH is safe in breastfeeding. | Greer IA, Nelson-Piercy C. Low-molecular-weight heparins for thromboprophylaxis and treatment of venous thromboembolism in pregnancy: a systematic review of safety and efficacy. Blood 2005;106:401–7. |  | PRE SPECIFIED ANALYSIS , STATISTICAL METHODS |
| Management of Beta Thalassaemia in Pregnancy | Folic acid (5 mg) is recommended preconceptually to all women to prevent neural tube defects. | Guideline Development Group. Management of diabetes from preconception to the postnatal period: summary of NICE guidance. BMJ 2008;336:714–7. |  |  |
|  |  | MRC Vitamin Study Research Group. Prevention of neural tube defects: results of the Medical Research Council Vitamin Study. Lancet 1991;ii:131–7.rct | AGE | RANDOMISATION, MASKING, ALLOCATION CONCEALMENT , PRE SPECIFIED ANALYSIS , STOPPING RULES , BASELINE DATA |
|  | Active management of the third stage of labour is recommended to minimise blood loss. | Royal College of Obstetricians and Gynaecologists. Blood Transfusions in Obstetrics. Green-top Guideline No. 47.London: RCOG; 2007. |  |  |
|  |  | Begley CM, Gyte GM, Devane D, McGuire W, Weeks A. Active versus expectant management for women in the third stage of labour. Cochrane Database Syst Rev 2011;(11):CD007412. |  | RANDOMISATION, MASKING, ALLOCATION CONCEALMENT , HANDLING OF WITHDRAWAL AND DROPOUTS , MEASURES OF VARIABILITY , PRE SPECIFIED ANALYSIS , STATISTICAL METHODS |
| Prevention and Mx of PPH | Uterine massage is of no benefit in the prophylaxis of PPH. | Hofmeyr GJ, Abdel-Aleem H, Abdel-Aleem MA. Uterine massagefor preventing postpartum haemorrhage. Cochrane Database SystRev 2013;(7):CD006431 |  | RANDOMISATION, MASKING, ALLOCATION CONCEALMENT , HANDLING OF WITHDRAWAL AND DROPOUTS , PRE SPECIFIED ANALYSIS , STATISTICAL METHODS |
|  | Prophylactic uterotonics should be routinely offered during the third stage of labour. | Hofmeyr GJ, Abdel-Aleem H, Abdel-Aleem MA. Uterine massagefor preventing postpartum haemorrhage. Cochrane Database SystRev 2013;(7):CD006431. |  | RANDOMISATION, MASKING, ALLOCATION CONCEALMENT , HANDLING OF WITHDRAWAL AND DROPOUTS , PRE SPECIFIED ANALYSIS , STATISTICAL METHODS |
|  |  | McDonald SJ, Abbott JM, Higgins SP. Prophylactic ergometrine-oxytocin versus oxytocin for the third stage of labour.Cochrane Database Syst Rev 2004;(1):CD000201.37. |  | RANDOMISATION, ALLOCATION CONCEALMENT , PRE SPECIFIED ANALYSIS , STATISTICAL METHODS |
|  |  | Tuncßalp €O, Hofmeyr GJ, G€ulmezoglu AM. Prostaglandins forpreventing postpartum haemorrhage. Cochrane Database Syst Rev2012;(8):CD000494. |  | RANDOMISATION, MASKING, ALLOCATION CONCEALMENT , HANDLING OF WITHDRAWAL AND DROPOUTS , MEASURES OF VARIABILITY , PRE SPECIFIED ANALYSIS , STATISTICAL METHODS , BASELINE DATA |
|  |  | Oladapo OT, Fawole B, Blum J, Abalos E. Advance misoprostoldistribution for preventing and treating postpartum haemorrhage.Cochrane Database Syst Rev 2012;(2):CD009336. |  |  |
|  | Oxytocin 10 iu IM is the agent of choice for PPH prophylaxis in vaginal delivery. | Tita AT, Szychowski JM, Rouse DJ, Bean CM, Chapman V,Nothern A, et al. Higher-dose oxytocin and hemorrhage aftervaginal delivery: a randomized controlled trial. Obstet Gynecol2012;119:293–300 | AGE , RACE, ETHNICITY AND CULTURE , BMI | RANDOMISATION, MASKING, ALLOCATION CONCEALMENT , PRE SPECIFIED ANALYSIS , STOPPING RULES , STATISTICAL METHODS , BASELINE DATA |
|  | Consider IV tranexamic acid (0.5–1.0 g) with oxytocin in women at increased PPH risk at caesarean. | Novikova N, Hofmeyr GJ, Cluver C. Tranexamic acid forpreventing postpartum haemorrhage. Cochrane Database Syst Rev2015;(6):CD007872 | BMI | RANDOMISATION, MASKING, ALLOCATION CONCEALMENT , HANDLING OF WITHDRAWAL AND DROPOUTS , PRE SPECIFIED ANALYSIS , STATISTICAL METHODS , ADDRESS MULTIPLICITY |
| Mx of Monochorionic Twin Pregnancy | cff DNA testing (NIPT) contingent on the first-trimester combined test is recommended for twin pregnancies including monochorionic twins. | H. Judah, M. M. Gil, A. Syngelaki, et al., “Cell-Free DNA Testingof Maternal Blood in Screening for Trisomies in Twin Pregnancy:Updated Cohort Study at 10-14 Weeks and Meta-Analysis,” Ultrasoundin Obstetrics & Gynecology 58, no. 2 (2021): 178–189. | AGE | MEASURES OF VARIABILITY , PRE SPECIFIED ANALYSIS , STATISTICAL METHODS , BASELINE DATA |
|  |  | M. M. Gil, R. Akolekar, M. S. Quezada, B. Bregant, and K. H.Nicolaides, “Analysis of Cell-Free DNA in Maternal Blood in Screeningfor Aneuploidies: Meta-Analysis,” Fetal Diagnosis and Therapy 35(2014): 156–173 |  | PRE SPECIFIED ANALYSIS , STATISTICAL METHODS , ADDRESS MULTIPLICITY |
|  | TTTS before 26 weeks should be treated by fetoscopic laser ablation rather than amnioreduction or septostomy. | M. V. Senat, J. Deprest, M. Boulvain, A. Paupe, N. Winer, and Y.Ville, “Endoscopic Laser Surgery Versus Serial Amnioreduction for Severe Twin-To-Twin Transfusion Syndrome,” New England Journal ofMedicine 351 (2004): 136–144.- RCT |  | RANDOMISATION, MEASURES OF VARIABILITY , PRE SPECIFIED ANALYSIS , STATISTICAL METHODS , ADDRESS MULTIPLICITY |
|  |  | K. J. Moise, Jr., K. Dorman, G. Lamvu, et al., “A Randomized Trialof Amnioreduction Versus Septostomy in the Treatment of Twin-TwinTransfusion Syndrome,” American Journal of Obstetrics and Gynecology193 (2005): 701–707. |  | RANDOMISATION, MEASURES OF VARIABILITY , PRE SPECIFIED ANALYSIS , STOPPING RULES , STATISTICAL METHODS , BASELINE DATA |
|  |  | T. M. Crombleholme, D. Shera, H. Lee, et al., “A Prospective,Randomized, Multicenter Trial of Amnioreduction vs SelectiveFetoscopic Laser Photocoagulation for the Treatment of Severe Twin-Twin Transfusion Syndrome,” American Journal of Obstetrics andGynecology 197, no. 396 (2007): e1–e9.94. |  | RANDOMISATION, HANDLING OF WITHDRAWAL AND DROPOUTS , PRE SPECIFIED ANALYSIS , STOPPING RULES , STATISTICAL METHODS , BASELINE DATA |
|  |  | D. Roberts, J. P. Neilson, M. D. Kilby, and S. Gates, “Interventionsfor the Treatment of Twin-Twin Transfusion Syndrome,” CochraneDatabase of Systematic Reviews 2014, no. 1 (2014): CD002073. |  | RANDOMISATION, MASKING, ALLOCATION CONCEALMENT , HANDLING OF WITHDRAWAL AND DROPOUTS , PRE SPECIFIED ANALYSIS , STATISTICAL METHODS , ADDRESS MULTIPLICITY |
|  |  | D. Murgano, A. Khalil, F. Prefumo, et al., “Outcome of Twin-To-Twin Transfusion Syndrome in Monochorionic Monoamniotic TwinPregnancy: Systematic Review and Meta-Analysis,” Ultrasound inObstetrics & Gynecology 55, no. 3 (2020): 310–317. |  | PRE SPECIFIED ANALYSIS , STATISTICAL METHODS , ADDRESS MULTIPLICITY |
|  |  | 96. J. Stirnemann, F. Slaghekke, N. Khalek, et al., “IntrauterineFetoscopic Laser Surgery Versus Expectant Management in Stage 1Twin-To-Twin Transfusion Syndrome: An International RandomizedTrial,” American Journal of Obstetrics and Gynecology 224, no. 5 (2021):528.e1–528.e12. | AGE , BMI | RANDOMISATION, HANDLING OF WITHDRAWAL AND DROPOUTS , MEASURES OF VARIABILITY , PRE SPECIFIED ANALYSIS , STATISTICAL METHODS , BASELINE DATA |
|  |  | F. Slaghekke, E. Lopriore, L. Lewi, et al., “Fetoscopic LaserCoagulation of the Vascular Equator Versus Selective Coagulation forTwin-To-Twin Transfusion Syndrome: An Open-Label RandomisedControlled Trial,” Lancet 383 (2014): 2144–2151 | AGE | RANDOMISATION, MASKING, ALLOCATION CONCEALMENT , MEASURES OF VARIABILITY , PRE SPECIFIED ANALYSIS , STATISTICAL METHODS , BASELINE DATA |
|  | Fetoscopic laser ablation should follow the serial, sequential SOlOMON technique. | D. Roberts, J. P. Neilson, M. D. Kilby, and S. Gates, “Interventionsfor the Treatment of Twin-Twin Transfusion Syndrome,” CochraneDatabase of Systematic Reviews 2014, no. 1 (2014): CD002073. |  | RANDOMISATION, MASKING, ALLOCATION CONCEALMENT , HANDLING OF WITHDRAWAL AND DROPOUTS , PRE SPECIFIED ANALYSIS , STATISTICAL METHODS , ADDRESS MULTIPLICITY |
|  |  | F. Slaghekke, E. Lopriore, L. Lewi, et al., “Fetoscopic LaserCoagulation of the Vascular Equator Versus Selective Coagulation forTwin-To-Twin Transfusion Syndrome: An Open-Label RandomisedControlled Trial,” Lancet 383 (2014): 2144–2151 | AGE | RANDOMISATION, MASKING, ALLOCATION CONCEALMENT , MEASURES OF VARIABILITY , PRE SPECIFIED ANALYSIS , STATISTICAL METHODS , BASELINE DATA |
|  | It is appropriate to aim for vaginal birth of MCDA twins unless there are other specific clinical indications. | J. F. Barrett, M. E. Hannah, E. K. Hutton, et al., “A RandomizedTrial of Planned Cesarean or Vaginal Delivery for Twin Pregnancy,”New England Journal of Medicine 369 (2013): 1295–1305 | AGE | RANDOMISATION, MASKING, STATISTICAL METHODS |
| Malaria in Pregnancy and Prevention | Inform women about bite prevention measures including repellents, insecticide-treated bed nets, knock-down sprays, protective clothing, and room protection. | McGready R, Hamilton KA, Simpson JA, Cho T, Luxemburger C,Edwards R, et al. Safety of the insect repellent N,N-diethyl-Mtoluamide (DEET) in pregnancy. Am J Trop Med Hyg. 2001;65:285–9.RCT | AGE | RANDOMISATION, PRE SPECIFIED ANALYSIS , STATISTICAL METHODS , BASELINE DATA |
|  |  | McGready R, Simpson JA, Htway M, White NJ, Nosten F, Lindsay SW. A double-blind randomized therapeutic trial of insect repellents for the prevention of malaria in pregnancy. Trans R Soc Trop Med Hyg 2001;95: | AGE , VULNERABLE | RANDOMISATION, MASKING, MEASURES OF VARIABILITY , PRE SPECIFIED ANALYSIS , BASELINE DATA |
|  |  | 37–8.Uzzan B, Konate L, Diop A, Nicolas P, Dia I, Dieng Y, et al. Efficacy of four insect repellents against mosquito bites: a double-blind randomized placebo-controlled field study in Senegal. Fundam Clin Pharmacol 2009;23:589–94. | AGE | RANDOMISATION, MASKING, MEASURES OF VARIABILITY , PRE SPECIFIED ANALYSIS , STATISTICAL METHODS , BASELINE DATA |
|  | Inform women (and their GP) of the risks and benefits of chemoprophylaxis versus malaria. | Hogh B, Clarke PD, Camus D, Nothdurft HD, Overbosch D, Gunther M, et al. Atovaquone-proguanil versus chloroquineproguanil for malaria prophylaxis in non-immune travellers: a randomised, double-blind study. Malarone International Study Team. Lancet 2000;356:1888–94. | AGE , RACE, ETHNICITY AND CULTURE | RANDOMISATION, MASKING, ALLOCATION CONCEALMENT , MEASURES OF VARIABILITY , PRE SPECIFIED ANALYSIS , STATISTICAL METHODS , BASELINE DATA, ADDRESS MULTIPLICITY |
|  |  | Nosten F, ter Kuile F, Maelankiri L, Chongsuphajaisiddhi T,Nopdonrattakoon L, Tangkitchot S, et al. Mefloquine prophylaxis prevents malaria during pregnancy: a double-blind,placebo-controlled study. J Infect Dis 1994;169:595–603. | AGE , VULNERABLE | RANDOMISATION, MASKING, MEASURES OF VARIABILITY , PRE SPECIFIED ANALYSIS , STATISTICAL METHODS , BASELINE DATA |
|  |  | Briand V, Bottero J, Noel H, Masse V, Cordel H, Guerra J, et al. Intermittent treatment for the prevention of malaria during pregnancy in Benin: a randomized, open-label equivalence trial comparing sulfadoxine–pyrimethamine with mefloquine. J Infect Dis 2009;200:991–1001. | AGE , LANGUAGE , EDUCATION, SOCIOECONOMIC, RESIDENCE, BMI | RANDOMISATION, HANDLING OF WITHDRAWAL AND DROPOUTS , MEASURES OF VARIABILITY , PRE SPECIFIED ANALYSIS , STATISTICAL METHODS , BASELINE DATA |
|  |  | Harinasuta T, Kietinun S, Somlaw S, Bunnag D, Sheth UK,Wernsdorfer W. A clinical trial of mefloquine on multi-resistant falciparum malaria in pregnant women in Thailand. Bull Soc Fr Parasitol 1990:429. | BMI | RANDOMISATION, MASKING, ALLOCATION CONCEALMENT , MEASURES OF VARIABILITY , PRE SPECIFIED ANALYSIS , BASELINE DATA |
|  |  | McGready R, Brockman A, Cho T, Cho D, van Vugt M,Luxemburger C, et al. Randomized comparison of mefloquineartesunate versus quinine in the treatment of multidrugresistant falciparum malaria in pregnancy. Trans R Soc Trop Med Hyg 2000;94:689–93. | AGE , LANGUAGE , VULNERABLE | RANDOMISATION, HANDLING OF WITHDRAWAL AND DROPOUTS , MEASURES OF VARIABILITY , PRE SPECIFIED ANALYSIS , STOPPING RULES , STATISTICAL METHODS , BASELINE DATA |
|  |  | McGready R, Cho T, Hkirijaroen L, Simpson J,Chongsuphajaisiddhi T, White NJ, et al. Quinine and mefloquine in the treatment of multidrug-resistant Plasmodium falciparum malaria in pregnancy. Ann Trop Med Parasitol1998;92:643–53. RCT. | AGE , VULNERABLE | MEASURES OF VARIABILITY , PRE SPECIFIED ANALYSIS , STATISTICAL METHODS , BASELINE DATA |
|  |  | Harinasuta T, Kietinun S, Somlaw S, Bunnag D, Sheth UK, Wernsdorfer W. A clinical trial of mefloquine on multi–resistant falciparum malaria in pregnant women in Thailand. Bulletin dela Societe Francaise de Parasitologie 1990:429. | BMI | RANDOMISATION, MASKING, ALLOCATION CONCEALMENT , MEASURES OF VARIABILITY , PRE SPECIFIED ANALYSIS , BASELINE DATA |
|  | Remind women that no malaria prophylaxis regimen is 100% protective. | Hogh B, Clarke PD, Camus D, Nothdurft HD, Overbosch D, Gunther M, et al. Atovaquone-proguanil versus chloroquineproguanil for malaria prophylaxis in non-immune travellers: a randomised, double-blind study. Malarone International Study Team. Lancet 2000;356:1888–94. | AGE , RACE, ETHNICITY AND CULTURE | RANDOMISATION, MASKING, ALLOCATION CONCEALMENT , MEASURES OF VARIABILITY , PRE SPECIFIED ANALYSIS , STATISTICAL METHODS , BASELINE DATA, ADDRESS MULTIPLICITY |
| [The Diagnosis and Treatment of Malaria in Pregnancy](https://www.rcog.org.uk/guidance/browse-all-guidance/green-top-guidelines/the-diagnosis-and-treatment-of-malaria-in-pregnancy-green-top-guideline-no-54b/) | Microscopic diagnosis allows species identification and estimation of parasitaemia, enabling appropriate treatment. | Chilton D, Malik AN, Armstrong M, Kettelhut M, Parker-Williams J, Chiodini PL. Use of rapid diagnostic tests for diagnosis of malaria in the UK. J Clin Pathol 2006;59:862–6. BOTH observational study |  |  |
|  |  | 46. Ashley EA, Touabi M, Ahrer M, Hutagalung R, Htun K, Luchavez J, et al. Evaluation of three parasite lactate dehydrogenase–based rapid diagnostic tests for the diagnosis of falciparum and vivax malaria. Malar J 2009;8:241. - | AGE , LANGUAGE | MASKING, ALLOCATION CONCEALMENT , MEASURES OF VARIABILITY , PRE SPECIFIED ANALYSIS , STOPPING RULES , STATISTICAL METHODS , BASELINE DATA |
|  | Admit all pregnant women with uncomplicated malaria to hospital; severe cases to intensive care. | Lalloo DG, Shingadia D, Pasvol G, Chiodini PL, Whitty CJ, Beeching NJ, et al. UK malaria treatment guidelines. J Infect 2007;54:111–21. | AGE |  |
|  | Use IV artesunate for severe falciparum malaria; IV quinine if artesunate unavailable. | Lalloo DG, Shingadia D, Pasvol G, Chiodini PL, Whitty CJ, Beeching NJ, et al. UK malaria treatment guidelines. J Infect 2007;54:111–21. | AGE |  |
|  | Use chloroquine to treat P. vivax, P. ovale, and P. malariae. | Lalloo DG, Shingadia D, Pasvol G, Chiodini PL, Whitty CJ, Beeching NJ, et al. UK malaria treatment guidelines. J Infect 2007;54:111–21. | AGE |  |
|  | Screen and treat for anaemia in women with malaria. | McGready R, Cho T, Keo NK, Thwai KL, Villegas L, Looareesuwan S, et al. Artemisinin antimalarials in pregnancy: a prospective treatment study of 539 episodes of multidrugresistant Plasmodium falciparum. Clin Infect Dis 2001;33:2009–16.prospective | AGE , VULNERABLE , MIGRANTS | MEASURES OF VARIABILITY , PRE SPECIFIED ANALYSIS , STOPPING RULES , STATISTICAL METHODS , BASELINE DATA |
|  | Uncomplicated malaria in pregnancy is not an indication for induction of labour. | Bounyasong S. Randomized trial of artesunate and mefloquine in comparison with quinine sulfate to treat P. falciparum malaria pregnant women. J Med Assoc Thai 2001;84:1289–99. |  |  |
|  |  | McGready R, Ashley EA, Moo E, Cho T, Barends M, Hutagalung R, et al. A randomized comparison of artesunate-atovaquoneproguanil versus quinine in treatment for uncomplicated falciparum malaria during pregnancy. J Infect Dis 2005;192:846–53. | AGE , LANGUAGE , VULNERABLE , MIGRANTS | RANDOMISATION, ALLOCATION CONCEALMENT , MEASURES OF VARIABILITY , PRE SPECIFIED ANALYSIS , STOPPING RULES , STATISTICAL METHODS , BASELINE DATA |
|  |  | McGready R, Brockman A, Cho T, Cho D, van Vugt M, Luxemburger C, et al. Randomized comparison of mefloquineartesunate versus quinine in the treatment of multidrugresistant falciparum malaria in pregnancy. Trans R Soc Trop Med Hyg 2000;94:689–93. | AGE , LANGUAGE | RANDOMISATION, MEASURES OF VARIABILITY , PRE SPECIFIED ANALYSIS , STOPPING RULES , STATISTICAL METHODS , BASELINE DATA |
|  |  | McGready R, Cho T, Samuel, Villegas L, Brockman A, van Vugt M,et al. Randomized comparison of quinine–clindamycin versus artesunate in the treatment of falciparum malaria in pregnancy. Trans R Soc Trop Med Hyg 2001;95:651–6. | AGE , LANGUAGE | RANDOMISATION, MEASURES OF VARIABILITY , PRE SPECIFIED ANALYSIS , STOPPING RULES , STATISTICAL METHODS , BASELINE DATA |
|  |  | Schultz LJ, Steketee RW, Macheso A, Kazembe P, Chitsulo L, Wirima JJ. The efficacy of antimalarial regimens containing sulfadoxine-pyrimethamine and/or chloroquine in preventing peripheral and placental Plasmodium falciparum infection among pregnant women in Malawi. Am J Trop Med Hyg 1994;51:515–22.RCT. |  |  |
|  |  | Verhoeff FH, Brabin BJ, Chimsuku L, Kazembe P, Russell WB, Broadhead RL. An evaluation of the effects of intermittent sulfadoxine–pyrimethamine treatment in pregnancy on parasite clearance and risk of low birthweight in rural Malawi. Ann Trop Med Parasitol 1998;92:141–50. RCT | AGE | MEASURES OF VARIABILITY , PRE SPECIFIED ANALYSIS , STOPPING RULES , STATISTICAL METHODS , BASELINE DATA |
| [Management of Inherited Bleeding Disorders in Pregnancy](https://www.rcog.org.uk/guidance/browse-all-guidance/green-top-guidelines/management-of-inherited-bleeding-disorders-in-pregnancy-green-top-guideline-no-71/) | The incidence in the non-Jewish population is 1 in 1,000,000; higher in Ashkenazi Jews (heterozygosity 8–9%, homozygosity 0.2–0.5%). | Peyvandi F, Duga S, Akhavan S, Mannucci PM. Rare coagulation deficiencies. Haemophilia 2002;8:308–21 - EPIDEMIOLOGICAL DATA. . |  |  |
|  |  | Seligsohn U. Factor XI deficiency. Thromb Haemost 1993;70:68–71 | AGE |  |
|  |  | Seligsohn U. High gene frequency of factor XI (PTA) deficiency in Ashkenazi Jews. Blood 1978;51:1223–8. | RACE, ETHNICITY AND CULTURE | MEASURES OF VARIABILITY , PRE SPECIFIED ANALYSIS , BASELINE DATA |
|  |  | Shpilberg O, Peretz H, Zivelin A, Yatuv R, Chetrit A, Kulka T,et al. One of the two common mutations causing factor XI deficiency in Ashkenazi Jews (type II) is also prevalent in Iraqi Jews, who represent the ancient gene pool of Jews. Blood 1995;85:429–32. - observational studies | AGE , RACE, ETHNICITY AND CULTURE | PRE SPECIFIED ANALYSIS , STATISTICAL METHODS |
|  | BSS is associated with high risk of primary and secondary PPH and wound haematoma. Delivery planning requires a multidisciplinary team. | Peitsidis P, Datta T, Pafilis I, Otomewo O, Tuddenham EG, Kadir RA. Bernard Soulier syndrome in pregnancy: a systematic review. Haemophilia 2010;16:584–91. | AGE , RACE, ETHNICITY AND CULTURE | MEASURES OF VARIABILITY , BASELINE DATA |
| [Prevention of Early-onset Group B Streptococcal Disease](https://www.rcog.org.uk/guidance/browse-all-guidance/green-top-guidelines/prevention-of-early-onset-group-b-streptococcal-disease-green-top-guideline-no-36/) | In women with negative or unknown GBS status, offer induction of labour immediately or expectant management up to 24 hours. Beyond 24 hours, induction is appropriate. | Seaward PG, Hannah ME, Myhr TL, Farine D, Ohlsson A, Wang EE,et al. International multicenter term PROM study: evaluation ofpredictors of neonatal infection in infants born to patients withpremature rupture of membranes at term. Premature Rupture ofthe Membranes. Am J Obstet Gynecol 1998;179:635–9. secondary analysis of a randomized controlled trial | AGE , DISABILITY - (PHYSICAL,ITELLECTUAL,MENTAL, SUBSTANCE MISUSE) | RANDOMISATION, MASKING, MEASURES OF VARIABILITY , PRE SPECIFIED ANALYSIS , STATISTICAL METHODS , BASELINE DATA |
|  |  | National Institute for Health and Clinical Excellence. Inducinglabour. NICE clinical guideline 70. London: NICE; 2008 |  |  |
| [Care of Women with Obesity in Pregnancy](https://www.rcog.org.uk/guidance/browse-all-guidance/green-top-guidelines/care-of-women-with-obesity-in-pregnancy-green-top-guideline-no-72/) | Active management of the third stage of labour should be recommended to reduce the risk of PPH. | Mavrides E, Allard S, Chandraharan E, Collins P, Green L, Hunt BJ,et al. Prevention and management of postpartum haemorrhage.BJOG 2016;124:e106–49. GUIDELINE. |  |  |
|  |  | Sebire NJ, Jolly M, Harris JP, Wadsworth J, Joffe M, Beard RW,et al. Maternal obesity and pregnancy outcome: a study of287,213 pregnancies in London. Int J Obes Relat Metab Disord2001;25:1175–82. RETROSPECTIVE COHORT STUDY | AGE , RACE, ETHNICITY AND CULTURE , BMI | MEASURES OF VARIABILITY , PRE SPECIFIED ANALYSIS , STOPPING RULES , BASELINE DATA |
|  | Women with class I obesity or greater having a caesarean section should receive prophylactic antibiotics at the time of surgery. | Smaill FM, Grivell RM. Antibiotic prophylaxis versus noprophylaxis for preventing infection after cesarean section.Cochrane Database Syst Rev 2014;(10):CD007482 | SOCIOECONOMIC | RANDOMISATION, MASKING, ALLOCATION CONCEALMENT , HANDLING OF WITHDRAWAL AND DROPOUTS , MEASURES OF VARIABILITY , PRE SPECIFIED ANALYSIS , STATISTICAL METHODS |
|  | Women undergoing caesarean section with >2 cm subcutaneous fat should have suturing of the tissue space to reduce wound infection and separation. | National Institute of Health and Care Excellence. CaesareanSection. Clinical Guideline/Meta-analysis 132. Manchester: NICE; 2011. | DISABILITY - (PHYSICAL,ITELLECTUAL,MENTAL, SUBSTANCE MISUSE), LANGUAGE , BMI |  |
|  |  | Allaire AD, Fisch J, McMahon MJ. Subcutaneous drain vs. suturein obese women undergoing cesarean delivery. A prospective,randomized trial. J Reprod Med 2000;45:327–31. |  |  |
|  |  | Cetin A, Cetin M. Superﬁcial wound disruption after cesareandelivery: effect of the depth and closure of subcutaneous tissue.Int J Gynaecol Obstet 1997;57:17–21. RCT | AGE , BMI | RANDOMISATION, MEASURES OF VARIABILITY , PRE SPECIFIED ANALYSIS , STOPPING RULES , STATISTICAL METHODS , BASELINE DATA |
| Blood Transfusion in Obstetrics | Oral iron should be the preferred first-line treatment for iron deficiency. | Haider BA, Olofin I, Wang M, Spiegelman D, Ezzati M, Fawzi WW. Anaemia, prenatal iron use, and risk of adverse pregnancy outcomes: systematic review and meta-analysis. BMJ 2013;346:f3443. |  | RANDOMISATION, MASKING, ALLOCATION CONCEALMENT , HANDLING OF WITHDRAWAL AND DROPOUTS , MEASURES OF VARIABILITY , PRE SPECIFIED ANALYSIS , STATISTICAL METHODS , BASELINE DATA |
|  |  | Pena-Rosas JP, De-Regil LM, Dowswell T, Viteri FE. Dailyoral iron supplementation during pregnancy. Cochrane Database Syst Rev 2012;(12):CD004736. |  | RANDOMISATION, MASKING, ALLOCATION CONCEALMENT , HANDLING OF WITHDRAWAL AND DROPOUTS , MEASURES OF VARIABILITY , PRE SPECIFIED ANALYSIS , STATISTICAL METHODS |
|  |  | Pena-Rosas JP, De-Regil LM, Dowswell T, Viteri FE.Intermittent oral iron supplementation during pregnancy.Cochrane Database Syst Rev 2012;(7):CD009997 |  | RANDOMISATION, MASKING, ALLOCATION CONCEALMENT , HANDLING OF WITHDRAWAL AND DROPOUTS , PRE SPECIFIED ANALYSIS , STATISTICAL METHODS |
|  | Active management of the third stage of labour should be recommended to reduce blood loss. | Prendiville WJ, Harding JE, Elbourne DR, Stirrat GM. The Bristol third stage trial: active versus physiological management of third stage of labour. BMJ 1988;297:1295–1300. RCT | AGE | RANDOMISATION, MASKING, HANDLING OF WITHDRAWAL AND DROPOUTS , MEASURES OF VARIABILITY , PRE SPECIFIED ANALYSIS , STOPPING RULES , STATISTICAL METHODS , BASELINE DATA |
|  |  | Prendiville WJ, Elbourne D, McDonald S. Active versus expectant management in the third stage of labour.Cochrane Database Syst Rev 2000;(2):CD000007.20. |  | RANDOMISATION |
|  |  | Rogers - Active vs Expectant Mx of 3rd Stage of Labour - Hichinbrooke Randomised | AGE , DISABILITY - (PHYSICAL,ITELLECTUAL,MENTAL, SUBSTANCE MISUSE) | RANDOMISATION, MASKING, MEASURES OF VARIABILITY , PRE SPECIFIED ANALYSIS , STOPPING RULES , STATISTICAL METHODS , BASELINE DATA |
| [Birth after Previous Caesarean Birth](https://www.rcog.org.uk/guidance/browse-all-guidance/green-top-guidelines/birth-after-previous-caesarean-birth-green-top-guideline-no-45/) | ERCS delivery should be conducted after 39+0 weeks of gestation. | Go MD, Emeis C, Guise JM, Schelonka RL. Fetal and neonatal morbidity and mortality following delivery after previous cesarean. Clin Perinatol 2011;38:311–9.SYS RV. |  |  |
|  |  | National Institute for Health and Clinical Excellence.Caesarean section. NICE clinical guideline. 132.Manchester: NICE; 2011. | DISABILITY - (PHYSICAL,ITELLECTUAL,MENTAL, SUBSTANCE MISUSE), LANGUAGE , BMI |  |
|  |  | Antenatal Corticosteroids to Reduce Neonatal Morbidity and Mortality. Green-top Guideline No. 7. London: RCOG;2010. |  |  |
|  |  | Stutchfield P, Whitaker R, Russell I; Antenatal Steroids for Term Elective Caesarean Section (ASTECS) Research Team. Antenatal betamethasone and incidence of neonatal respiratory distress after elective caesarean section: pragmatic randomised trial. BMJ 2005;331:662. | AGE , RACE, ETHNICITY AND CULTURE | RANDOMISATION, MEASURES OF VARIABILITY , PRE SPECIFIED ANALYSIS , STOPPING RULES , STATISTICAL METHODS , BASELINE DATA |
|  |  | Stutchfield PR, Whitaker R, Gliddon AE, Hobson L, Kotecha S, Doull IJ. Behavioural, educational and respiratory outcomes of antenatal betamethasone for term caesarean section (ASTECS trial). Arch Dis Child Fetal Neonatal Ed 2013;98:F195–200.F/U RCT. | AGE |  |
|  |  | Kenyon SL, Taylor DJ, Tarnow-Mordi W; ORACLE Collaborative Group. Broad-spectrum antibiotics for preterm, prelabour rupture of fetal membranes: the ORACLE I randomised trial. Lancet 2001;357:979–88. | AGE | RANDOMISATION, MASKING, ALLOCATION CONCEALMENT , MEASURES OF VARIABILITY , PRE SPECIFIED ANALYSIS , STOPPING RULES , STATISTICAL METHODS , BASELINE DATA |
|  |  | Kenyon SL, Taylor DJ, Tarnow-Mordi W; ORACLE Collaborative Group. Broad-spectrum antibiotics for spontaneous preterm labour: the ORACLE II randomised trial. Lancet 2001;357:989–94. | AGE | RANDOMISATION, MASKING, ALLOCATION CONCEALMENT , HANDLING OF WITHDRAWAL AND DROPOUTS , MEASURES OF VARIABILITY , PRE SPECIFIED ANALYSIS , STOPPING RULES , STATISTICAL METHODS , BASELINE DATA |
| Assisted Vaginal Birth | Encourage women to have continuous support during labour to reduce the need for assisted vaginal birth. | Bohren MA, Hofmeyr GJ, Sakala C, Fukuzawa RK, Cuthbert A.Continuous support for women during childbirth. CochraneDatabase of Syst Rev 2017;7:CD003766. | SOCIOECONOMIC | RANDOMISATION, MASKING, ALLOCATION CONCEALMENT , HANDLING OF WITHDRAWAL AND DROPOUTS , MEASURES OF VARIABILITY , PRE SPECIFIED ANALYSIS , STATISTICAL METHODS |
|  | Inform women that epidural analgesia may increase the need for assisted vaginal birth, though this is less likely with modern techniques. | Anim-Somuah M, Smyth RM, Jones L. Epidural versus non-epidural or no analgesia in labour. Cochrane Database Syst Rev2011;12:CD000331. Update 2018 |  | RANDOMISATION, HANDLING OF WITHDRAWAL AND DROPOUTS , PRE SPECIFIED ANALYSIS , STATISTICAL METHODS |
|  | Administering epidural in the latent phase does not increase assisted birth risk compared to active phase. | Wassen MM, Zuijlen J, Roumen FJ, Smits LJ, Marcus MA, Nijhuis JG.Early versus late epidural analgesia and risk of instrumental deliveryin nulliparous women: a systematic review. BJOG 2011;118:655–61 |  | RANDOMISATION, HANDLING OF WITHDRAWAL AND DROPOUTS , MEASURES OF VARIABILITY , PRE SPECIFIED ANALYSIS , STOPPING RULES , STATISTICAL METHODS |
|  | Encourage upright or lateral positions in second stage for women without epidurals. | Gupta JK, Sood A, Hofmeyr GJ, Vogel JP. Position in the secondstage of labour for women without epidural analgesia. CochraneDatabase Syst Rev 2017;5:CD002006. |  | RANDOMISATION, HANDLING OF WITHDRAWAL AND DROPOUTS , MEASURES OF VARIABILITY , PRE SPECIFIED ANALYSIS , STATISTICAL METHODS |
|  | Women with epidurals should adopt lying down lateral positions in second stage to increase spontaneous birth. | Epidural and Position Trial Collaborative Group. Upright versuslying down position in second stage of labour in nulliparouswomen with low dose epidural: BUMPES randomised controlledtrial. BMJ 2017;359:j4471. | AGE , RACE, ETHNICITY AND CULTURE , SOCIOECONOMIC, BMI | RANDOMISATION, HANDLING OF WITHDRAWAL AND DROPOUTS , MEASURES OF VARIABILITY , PRE SPECIFIED ANALYSIS , STOPPING RULES , STATISTICAL METHODS , BASELINE DATA, ADDRESS MULTIPLICITY |
|  |  | Kibuka M, Thornton JG. Position in second stage of labour forwomen with epidural analgesia. Cochrane Database Syst Rev2017;2:CD008070 |  | RANDOMISATION, MASKING, ALLOCATION CONCEALMENT , HANDLING OF WITHDRAWAL AND DROPOUTS , MEASURES OF VARIABILITY , PRE SPECIFIED ANALYSIS , STATISTICAL METHODS , ADDRESS MULTIPLICITY |
|  | Do not discontinue epidural during pushing. | Torvaldsen S, Roberts CL, Bell JC, Raynes-Greenow CH.Discontinuation of epidural analgesia late in labour for reducingthe adverse delivery outcomes associated with epidural analgesia.Cochrane Database Syst Rev 2004;4:CD004457. |  | RANDOMISATION, MASKING, ALLOCATION CONCEALMENT , MEASURES OF VARIABILITY , PRE SPECIFIED ANALYSIS , ADDRESS MULTIPLICITY |
|  | Do not recommend any specific regional technique to reduce assisted vaginal birth incidence. | Simmons SW, Cyna AM, Dennis AT, Hughes D. Combinedspinal-epidural versus epidural analgesia in labour. CochraneDatabase Syst Rev 2007;3:CD003401. |  | RANDOMISATION, MASKING, ALLOCATION CONCEALMENT , MEASURES OF VARIABILITY , PRE SPECIFIED ANALYSIS , STATISTICAL METHODS , BASELINE DATA, ADDRESS MULTIPLICITY |
|  |  | van der Vyver M, Halpern S, Joseph G. Patient-controlledepidural analgesia versus continuous infusion for labour analgesia:a meta-analysis. Br J Anaesth 2002;89:459–65. |  | RANDOMISATION, MASKING, MEASURES OF VARIABILITY , PRE SPECIFIED ANALYSIS , STATISTICAL METHODS |
|  |  | Wong CA, Ratliff JT, Sullivan JT, Scavone BM, Toledo P,McCarthy RJ. A randomized comparison of programmedintermittent epidural bolus with continuous epidural infusion forlabor analgesia. Anesth Analg 2006;102:904–9. | BMI | RANDOMISATION, MASKING, MEASURES OF VARIABILITY , PRE SPECIFIED ANALYSIS , STOPPING RULES , STATISTICAL METHODS , BASELINE DATA, ADDRESS MULTIPLICITY |
|  | Do not recommend routine oxytocin augmentation with epidural to reduce assisted birth. | Costley PL, East CE. Oxytocin augmentation of labour in womenwith epidural analgesia for reducing operative deliveries. CochraneDatabase Syst Rev 2013;7:CD009241.22. |  | RANDOMISATION, MASKING, ALLOCATION CONCEALMENT , HANDLING OF WITHDRAWAL AND DROPOUTS , PRE SPECIFIED ANALYSIS , STATISTICAL METHODS |
|  |  | National Institute for Health and Care Excellence. Intrapartumcare for healthy women and babies. Clinical Guideline 190. London:NICE; 2017 |  |  |
|  | Clinicians should be aware that ultrasound assessment of fetal head position prior to assisted vaginal birth is more reliable than clinical examination. | Ramphul M, Ooi PV, Burke G, Kennelly MM, Said SAT,Montgomery AA, et al. Instrumental delivery and ultrasound: amulticentre randomised controlled trial of ultrasound assessmentof the fetal head position versus standard care as an approach toprevent morbidity at instrumental delivery. BJOG 2014;121:1029–1038. | AGE , BMI | RANDOMISATION, MEASURES OF VARIABILITY , PRE SPECIFIED ANALYSIS , STOPPING RULES , STATISTICAL METHODS , BASELINE DATA |
|  |  | Popowski T, Porcher R, Fort J, Javoise S, Rozenberg P. Inﬂuenceof ultrasound determination of fetal head position on mode ofdelivery: a pragmatic randomized trial. Ultrasound Obstet Gynecol2015;46:520–5 | AGE | RANDOMISATION, ALLOCATION CONCEALMENT , MEASURES OF VARIABILITY , PRE SPECIFIED ANALYSIS , STOPPING RULES , STATISTICAL METHODS , BASELINE DATA |
|  | Forceps and vacuum each have distinct risks and benefits; vacuum is more likely to fail, forceps more likely to cause maternal trauma. | Johanson RB, Menon BK. Vacuum extraction versus forceps forassisted vaginal delivery. Cochrane Database Syst Rev 2000;2:CD000224. |  | RANDOMISATION, MASKING, HANDLING OF WITHDRAWAL AND DROPOUTS , PRE SPECIFIED ANALYSIS , STATISTICAL METHODS |
|  |  | Johanson RB, Heycock E, Carter J, Sultan AH, Walklate K, JonesPW. Maternal and child health after assisted vaginal delivery: ﬁve-year follow up of a randomised controlled study comparingforceps and ventouse. Br J Obstet Gynaecol 1999;106:544–9. | AGE | MEASURES OF VARIABILITY , PRE SPECIFIED ANALYSIS , STATISTICAL METHODS |
|  | Soft cup vacuum extractors have higher failure but lower neonatal scalp trauma than rigid cups. | Attilakos G, Sibanda T, Winter C, Johnson N, Draycott T. A randomised controlled trial of a new handheld vacuumextraction device. BJOG 2005;112:1510–5.86. | AGE | RANDOMISATION, HANDLING OF WITHDRAWAL AND DROPOUTS , MEASURES OF VARIABILITY , PRE SPECIFIED ANALYSIS , STATISTICAL METHODS , BASELINE DATA |
|  |  | Groom KM, Jones BA, Miller N, Paterson-Brown S. A prospective randomised controlled trial of the Kiwi Omnicup versus conventional ventouse cups for vacuum-assisted vaginaldelivery. BJOG 2006;113:183–9.87. | AGE | RANDOMISATION, ALLOCATION CONCEALMENT , HANDLING OF WITHDRAWAL AND DROPOUTS , PRE SPECIFIED ANALYSIS , STOPPING RULES , STATISTICAL METHODS |
|  |  | Equy V, David-Tchouda S, Dreyfus M, Riethmuller D, VendittelliF, Cabaud V, et al. Clinical impact of the disposable ventouseiCupâ versus a metallic vacuum cup: a multicenter randomized controlled trial. BMC Pregnancy Childbirth 2015;15:332. | AGE | RANDOMISATION, MEASURES OF VARIABILITY , PRE SPECIFIED ANALYSIS , STATISTICAL METHODS , BASELINE DATA |
|  |  | Mola GD, Kuk JM. A randomised controlled trial of twoinstruments for vacuum-assisted delivery (Vacca Re-Usable OmniCup and the Bird anterior and posterior cups) to compare failure rates, safety and use effectiveness. Aust N Z J ObstetGynaecol 2010;50:246–52. | AGE | RANDOMISATION, ALLOCATION CONCEALMENT , MEASURES OF VARIABILITY , PRE SPECIFIED ANALYSIS , STATISTICAL METHODS , BASELINE DATA |
|  |  | Johanson R, Menon V. Soft versus rigid vacuum extractor cupsfor assisted vaginal delivery. Cochrane Database of Syst Rev 2000;2:CD000446. |  | RANDOMISATION, ALLOCATION CONCEALMENT , HANDLING OF WITHDRAWAL AND DROPOUTS , PRE SPECIFIED ANALYSIS , STATISTICAL METHODS |
|  |  | O’Mahony F, Hofmeyr GJ. Menon V. Choice of instruments forassisted vaginal delivery. Cochrane Database of Syst Rev 2010;11:CD005455 |  | RANDOMISATION, MASKING, ALLOCATION CONCEALMENT , HANDLING OF WITHDRAWAL AND DROPOUTS , PRE SPECIFIED ANALYSIS , STATISTICAL METHODS |
|  | A single prophylactic dose of intravenous amoxicillin and clavulanic acid should be given following assisted vaginal birth. | Liabsuetrakul T, Choobun T, Peeyananjarassri K, Islam QM.Antibiotic prophylaxis for operative vaginal delivery. CochraneDatabase Syst Rev 2017;8:CD004455. |  | RANDOMISATION, MASKING, ALLOCATION CONCEALMENT , HANDLING OF WITHDRAWAL AND DROPOUTS , PRE SPECIFIED ANALYSIS , STATISTICAL METHODS |
|  |  | Bonet M, Ota E, Chibueze CE, Oladapo OT. Routine antibioticprophylaxis after normal vaginal birth for reducing maternalinfectious morbidity. Cochrane Database Syst Rev 2017;11:CD012137. |  | RANDOMISATION, MASKING, ALLOCATION CONCEALMENT , HANDLING OF WITHDRAWAL AND DROPOUTS , PRE SPECIFIED ANALYSIS , STATISTICAL METHODS , BASELINE DATA, ADDRESS MULTIPLICITY |
|  |  | Knight M, Chiocchia V, Partlett C, Rivero-Arias O, Hua X,Hinshaw K, et al. on behalf of the ANODE collaborative group*.Prophylactic antibiotics in the prevention of infection afteroperative vaginal delivery (ANODE): a multicentre randomisedcontrolled trial. Lancet 2019;393:2395–403 | AGE , RACE, ETHNICITY AND CULTURE , BMI | RANDOMISATION, MASKING, ALLOCATION CONCEALMENT , HANDLING OF WITHDRAWAL AND DROPOUTS , MEASURES OF VARIABILITY , PRE SPECIFIED ANALYSIS , STATISTICAL METHODS , BASELINE DATA |
|  | In the absence of contraindications, women should be offered regular NSAIDs and paracetamol after birth. | Nikpoor P, Bain E. Analgesia for forceps delivery. CochraneDatabase Syst Rev 2013;9:CD008878 |  | RANDOMISATION, MASKING, ALLOCATION CONCEALMENT , HANDLING OF WITHDRAWAL AND DROPOUTS , PRE SPECIFIED ANALYSIS , STATISTICAL METHODS |
| [Antenatal corticosteroids to reduce neonatal morbidity and mortality](https://www.rcog.org.uk/guidance/browse-all-guidance/green-top-guidelines/antenatal-corticosteroids-to-reduce-neonatal-morbidity-and-mortality-green-top-guideline-no-74/) | A course of antenatal corticosteroids given within 7 days prior to preterm birth reduces perinatal and neonatal death and respiratory distress syndrome. | McGoldrick E, Stewart F, Parker R, Dalziel SR. Antenatalcorticosteroids for accelerating fetal lung maturation for women atrisk of preterm birth. Cochrane Database Syst Rev 2021;12:CD004454. |  | RANDOMISATION, MASKING, ALLOCATION CONCEALMENT , HANDLING OF WITHDRAWAL AND DROPOUTS , MEASURES OF VARIABILITY , PRE SPECIFIED ANALYSIS , STATISTICAL METHODS , ADDRESS MULTIPLICITY |
|  | Corticosteroids should be offered to women between 24+0 and 34+6 weeks’ gestation in whom imminent preterm birth is anticipated. | National Institue for Health and Care Excellence. Preterm labour andbirth. NICE guideline NG25. London: NICE; 2015. updated 2019. | RACE, ETHNICITY AND CULTURE |  |
|  |  | McGoldrick E, Stewart F, Parker R, Dalziel SR. Antenatalcorticosteroids for accelerating fetal lung maturation for women atrisk of preterm birth. Cochrane Database Syst Rev 2021;12:CD004454. |  | RANDOMISATION, MASKING, ALLOCATION CONCEALMENT , HANDLING OF WITHDRAWAL AND DROPOUTS , MEASURES OF VARIABILITY , PRE SPECIFIED ANALYSIS , STATISTICAL METHODS , ADDRESS MULTIPLICITY |
|  | Antenatal corticosteroids should be offered to women with PPROM who are at increased risk of preterm birth. | Peaceman AM, Lai Y, Rouse DJ, Spong CY, Mercer BM, Varner MW,et al. Length of latency with preterm premature rupture ofmembranes before 32 weeks’ gestation. Am J Perinatol 2015;32:57– 62.SECONDARY ANALYSIS OF DATA FROM RCT | AGE , RACE, ETHNICITY AND CULTURE , BMI | RANDOMISATION, MEASURES OF VARIABILITY , PRE SPECIFIED ANALYSIS , STATISTICAL METHODS , BASELINE DATA |
| Management of Thyroid Disorders in Pregnancy | Women on levothyroxine therapy for hypothyroidism should be counselled to self-initiate an empirical increasein their dose of levothyroxineby approximately 25%–30% assoon as they have a positivepregnancy test. This may beachieved by either:– doubling the dose oflevothyroxine on two days ofeach week or– implementing a doseincrement of:• 25μg per day for womentaking 100μg or lesslevothyroxine daily• 50μg per day for womentaking greater than 100μglevothyroxine daily | L. Yassa, E. Marqusee, R. Fawcett, and E. K. Alexander, “ThyroidHormone Early Adjustment in Pregnancy (The THERAPY) Trial,”Journal of Clinical Endocrinology and Metabolism 95, no. 7 (2010):3234–3241, https://doi.org/10.1210/jc.2010-0013. | AGE | RANDOMISATION, MEASURES OF VARIABILITY , PRE SPECIFIED ANALYSIS , STATISTICAL METHODS , BASELINE DATA |
|  | For pregnantwomen treated withlevothyroxine for hypothyroidism,TSH and fT4concentrationsshould be checkedevery 4–6 weeksuntil 20 weeks ofgestation then onceagain at 28 weeks ofgestation | L. Yassa, E. Marqusee, R. Fawcett, and E. K. Alexander, “ThyroidHormone Early Adjustment in Pregnancy (The THERAPY) Trial,”Journal of Clinical Endocrinology and Metabolism 95, no. 7 (2010):3234–3241, https://doi.org/10.1210/jc.2010-0013. | AGE | RANDOMISATION, MEASURES OF VARIABILITY , PRE SPECIFIED ANALYSIS , STATISTICAL METHODS , BASELINE DATA |
|  | Levothyroxine treatment is not recommended for women with TPOAb in the absence of thyroid dysfucntion during pregnancy | R. K. Dhillon- Smith, L. J. Middleton, K. K. Sunner, et al.,“Levothyroxine in Women With Thyroid Peroxidase Antibodies Before Conception,” New England Journal of Medicine 380, no. 14 (2019): 1316–1325, https://doi.org/10.1056/NEJMoa1812537. | AGE , RACE, ETHNICITY AND CULTURE , BMI | RANDOMISATION, MASKING, ALLOCATION CONCEALMENT , HANDLING OF WITHDRAWAL AND DROPOUTS , PRE SPECIFIED ANALYSIS , STATISTICAL METHODS , BASELINE DATA, ADDRESS MULTIPLICITY |
|  |  | S. Nazarpour, F. Ramezani Tehrani, M. Simbar, M. Tohidi, H. Alavi Majd, and F. Azizi, “Effects of Levothyroxine Treatment on Pregnancy Outcomes in Pregnant Women With Autoimmune Thyroid Disease,” European Journal of Endocrinology 176, no. 2 (2017): 253–265, https:// doi.org/10.1530/EJE-16-0548. | AGE , EDUCATION, BMI | RANDOMISATION, MASKING, HANDLING OF WITHDRAWAL AND DROPOUTS , MEASURES OF VARIABILITY , PRE SPECIFIED ANALYSIS , STATISTICAL METHODS , BASELINE DATA, ADDRESS MULTIPLICITY |

Table S7: Underpinning studies for Grade A recommendations

| **Guideline Name** | **Median and range of health equity score per guideline** |
| --- | --- |
| Identification and management of maternal sepsis during and following pregnancy | 4 |
| Intrahepatic cholestasis of pregnancy | 3 (0-4) |
| Cervical Cerclage | 2.5 (2-3) |
| Malaria in Pregnancy, Prevention | 2 (1-2) |
| Investigation and Care of a Small-for-Gestational-Age Fetus and a Growth Restricted Fetus | 1.5 (0-3) |
| Care of Women with Obesity in Pregnancy | 1.5 (1-2) |
| Management of Inherited Bleeding Disorders | 1.5 (1-2) |
| The Diagnosis and Treatment of Malaria in Pregnancy | 1 (1-3) |
| Amniocentesis and Chorionic Villous sampling | 1 |
| Management of Thyroid Disorders in Pregnancy | 1 (1-3) |
| Birth after Previous Caesarean Section | 1 |
| Prevention of Early-onset Group B Streptococcal Disease | 1 |
| Antenatal corticosteroids to reduce neonatal morbidity and mortality | 0.5 (0-3) |
| Prevention and Management of Postpartum Haemorrhage | 0.5 (0-3) |
| Management of Breech Presentation | 0.5 (0-1) |
| Management of Monochorionic Twin Pregnancy | 0.5 (0-1) |
| The Management of Third- and Fourth-Degree Perineal Tears | 0.5 (0-1) |
| Blood Transfusion in Obstetrics | 0.5 (0-1) |
| Maternal Collapse in Pregnancy and the Puerperium | 0.5 (0-1) |
| Management of Beta Thalassaemia in Pregnancy | 0.25 (0-0.5) |
| Care of Women Presenting with Suspected Preterm Prelabour Rupture of Membranes from 24+0 Weeks of Gestation | 0 (All Zero) |
| Assisted Vaginal Birth | 0 (0-2) |
| External Cephalic Version and Reducing the Incidence of Term Breech Presentation | 0 (0-1.5) |
| The Management of Nausea and Vomiting in Pregnancy and Hyperemesis Gravidarum | 0 (All Zero) |
| Care of late intrauterine fetal death and stillbirth | 0 (0-3) |
| Antepartum haemorrhage | 0 (All Zero) |
| Reduced Fetal Movements | 0 |
| Reducing the Risk of VTE during Pregnancy and the Puerperium | 0 (All Zero) |

Table S8: Median health equity score for each guideline (guideline level). Ranges differ by level of aggregation (study-level vs guideline-level medians).

| **Guideline Name** | **Median and range of generalisability score per guideline** |
| --- | --- |
| Cervical Cerclage | 8 (7-9) |
| The Management of Third- and Fourth-Degree Perineal Tears | 7 (7,7) |
| Blood Transfusion in Obstetrics | 7 (7,7) |
| Care of Women Presenting with Suspected Preterm Prelabour Rupture of Membranes from 24+0 Weeks of Gestation | 6.5 (0-7) |
| Prevention and Management of Postpartum Haemorrhage | 6.5 (5-7) |
| Investigation and Care of a Small-for-Gestational-Age Fetus and a Growth Restricted Fetus | 6.5 (0-8) |
| Malaria in Pregnancy, Prevention | 6 (5-8) |
| Intrahepatic cholestasis of pregnancy | 6 (6-9) |
| Assisted Vaginal Birth | 6 (3-8) |
| External Cephalic Version and Reducing the Incidence of Term Breech Presentation | 6 (6-6.5) |
| The Management of Nausea and Vomiting in Pregnancy and Hyperemesis Gravidarum | 6 (3-8) |
| Management of Breech Presentation | 6 (5.5-6) |
| Antenatal corticosteroids to reduce neonatal morbidity and mortality | 5 (4-8) |
| Amniocentesis and Chorionic Villous sampling | 5 |
| Management of Thyroid Disorders in Pregnancy | 5 (5-8) |
| Maternal Collapse in Pregnancy and the Puerperium | 5 (2-8) |
| Management of Monochorionic Twin Pregnancy | 4.75 (3-7) |
| Management of Beta Thalassaemia in Pregnancy | 3.25 (3-3.5) |
| Prevention of Early-onset Group B Streptococcal Disease | 3 |
| Care of late intrauterine fetal death and stillbirth | 2.75 (1-8) |
| Reducing the Risk of VTE during Pregnancy and the Puerperium | 2.5 (2-3) |
| Care of Women with Obesity in Pregnancy | 2 (0-7) |
| Antepartum haemorrhage | 2 (0-4) |
| Reduced Fetal Movements | 2 |
| The Diagnosis and Treatment of Malaria in Pregnancy | 1.75 (0-5.5) |
| Management of Inherited Bleeding Disorders | 1.5 |
| Birth after Previous Caesarean Section | 0 |
| Identification and management of maternal sepsis during and following pregnancy | 0 |

Table S9: Median generalisability score for each guideline. Ranges differ by level of aggregation (study-level vs guideline-level medians).
